# Engram cell vulnerability in neurodegeneration: from mechanistic insights to therapeutic horizons

**DOI:** 10.3389/fnagi.2026.1873893

**Published:** 2026-09-03

**Authors:** Qingyu Bai, Yan Yan, Chao Zeng, Xue Li, Guihua Zeng, Aleena Saidova, Hui Li, Hong Qing

**Affiliations:** 1 Department of Biology, Shenzhen MSU-BIT University, Shenzhen, China; 2 The First Hospital of Hebei Medical University, Shijiazhuang, Hebei, China

**Keywords:** Alzheimer’s disease, complement, engram cells, Huntington’s disease, microglia, Parkinson’s disease

## Abstract

Neurodegenerative diseases such as Alzheimer’s disease (AD), Parkinson’s disease (PD), and Huntington’s disease (HD) share progressive memory impairment, yet their upstream pathologies differ. This review synthesizes evidence across these disorders to frame memory decline through engram ensembles and proposes an excitability-vulnerability tradeoff, in which the physiological requirements for engram function, including elevated excitability, synaptic plasticity, and coordinated network activity, also increase sensitivity to synaptic stress, network disruption, and inflammatory perturbations. We summarize disease-related pathological cascades. To date, AD has the most robust body of direct experimental evidence linking pathology to impaired engram function. Solid experimental findings demonstrate that amyloid-β (Aβ)/tau causes hippocampal engram inaccessibility in AD. For PD and HD, inferences drawn from synaptic and circuit dysfunction suggest that α-synuclein (α-syn) pathology and dopaminergic loss may disrupt striatal ensembles, and mutant huntingtin (mHTT) presumably impairs striatocortical coordination. We further integrate a shared amplification route in which microglial activation and complement-linked synaptic remodeling weaken memory-relevant connectivity and reduce cue-driven ensemble reinstatement. This mechanism is well validated in AD, while supporting evidence for PD and HD remains largely indirect. Finally, we outline a translational roadmap that integrates circuit-targeted delivery, engram-relevant clinical endpoints, and scalable neuromodulation, supported by human neuronal-glial molecular profiling to guide mechanism-informed combination strategies. Importantly, these multi-tiered therapeutic approaches act on the full spectrum of neural cells rather than only memory engram ensembles, and cognitive rescue arises from a multifactorial regulatory process involving both global neuroprotection and ensemble stabilization. We also systematically discuss key limitations of current engram research, including methodological heterogeneity in engram labelling tools, interspecies translational barriers, and substantial clinical obstacles for optogenetic and circuit-based therapeutic applications.

## 1 Introduction

Unraveling the neural substrates of learning and memory is a fundamental pursuit in neuroscience, with engram cells emerging as a cornerstone of contemporary memory research. The concept of the “engram” was first articulated by Richard Semon in the early 20th century, however, its experimental validation awaited nearly a century of technological and conceptual advancements. Seminal research led by Tonegawa and colleagues provided compelling evidence that memory can be traced to discrete neuronal ensembles, whose selective reactivation is sufficient to drive the encoding, storage, and retrieval of specific memory traces (Josselyn and Tonegawa, 2020; Tonegawa et al., 2015). This framework has not only reshaped mechanistic models of memory, but also opened a transformative avenue for interrogating cognitive decline in brain disorders.

Progressive memory impairment is a shared clinical feature of AD, PD, and HD. Comparative neuropsychological studies indicate that all three disorders exhibit significant deficits in explicit and episodic memory, although they differ in the specific profiles of new learning and memory performance (Pillon et al., 1993; Aretouli and Brandt, 2010). Despite this shared phenotype, the primary pathogenic mechanisms diverge: AD is primarily associated with Aβ and tau pathology, PD is characterized by α-syn pathology and dopaminergic degeneration, and HD is driven by mHTT-related cellular and synaptic dysfunction. Notably, memory decline can occur in the absence of widespread neuronal loss, which aligns with the early disruption of engram ensembles that facilitate memory encoding and retrieval. Of the three disorders discussed herein, AD provides the strongest experimental support for our engram-centered framework, with abundant preclinical and clinical data confirming early engram impairment. In AD, this phenomenon has been validated by multiple animal and human studies as a result of early engram disruption, consistent with the broader “silent engram” concept originally established in physiological memory studies and subsequently extended to AD models (Roy et al., 2016; Ryan et al., 2015). In PD and HD, impaired memory is inferred to arise from dysfunction of memory-related ensembles, based on observed synaptic and circuit deficits (Iemolo et al., 2023; Lahr et al., 2018). Furthermore, microglia-mediated neuroinflammation may exacerbate engram dysfunction across these diseases (Wang et al., 2020; Wilton et al., 2023; Stoll et al., 2024).

Recent evidence suggests that engrams may not be limited to neurons alone. While neuronal engrams have been extensively studied, research on astrocyte-derived functional ensembles (astroengrams) remains an emerging field. Existing preliminary findings shown that astrocytes can create learning-induced functional ensembles known as astroengrams, which interact bidirectionally with neuronal engrams and play a crucial role in memory recall. This perspective extends the engram concept to encompass a multicellular framework (Sánchez Romero and Navarrete, 2026). This broader perspective implies that memory deterioration could result from the synchronized malfunction of neuronal-glial ensembles rather than isolated neuronal impairments, thereby expanding the range of potential therapeutic interventions.

Despite significant advancements in identifying engram cells and developing tools for their labeling and manipulation, a comprehensive synthesis of the selective vulnerability of engram ensembles in neurodegenerative diseases and the translation of this vulnerability into actionable therapeutic strategies remains scarce (Josselyn and Tonegawa, 2020; Balmer et al., 2025; Lazarov et al., 2024). In addition, current engram research faces prominent challenges, including inconsistent labelling methodologies, interspecies gaps and multiple clinical hurdles for cutting-edge neuromodulation techniques such as optogenetics. This review aims to address this gap. We begin by examining disease-specific pathways of vulnerability, detailing how key pathogenic factors (e.g., Aβ, α-syn, mHTT) preferentially disrupt the synaptic and cellular programs that support engrams. Building on this mechanistic understanding, we propose a cross-disease framework that correlates pathology-associated engram vulnerability with a tiered therapeutic strategy, which includes upstream pathology attenuation, engram protection, and circuit restoration. It should be emphasized that these multi-tiered therapeutic approaches act on the full spectrum of neural cells rather than only memory engram ensembles, and cognitive rescue arises from a multifactorial regulatory process. Finally, drawing on insights from innovative methodologies such as optogenetics and single-cell/multi-omics profiling, we outline translational priorities for an engram-centered roadmap. This roadmap encompasses enhanced *in vivo* tracking of ensemble dynamics, multicellular (neuron-glia) engram biomarkers, and rational combinatorial interventions designed to preserve memory function.

## 2 Foundational mechanisms of engram cells: the basis for vulnerability

### 2.1 Definition and formation of engram cells

Engram cells are specialized neuronal assemblies that encode specific memories, offering a mechanistic framework for understanding selective memory disruption in neurodegenerative disorders. According to Josselyn and Tonegawa, engram cells are activated during memory acquisition, reactivated during retrieval, and exhibit functional specificity in associating an experience with subsequent recall (Josselyn and Tonegawa, 2020; Lau et al., 2020). Conceptually, memory processing encompasses engram formation, consolidation, retrieval, and forgetting. The allocation of engrams is influenced by intrinsic cellular properties. Neurons characterized by higher baseline excitability and enhanced synaptic connectivity are preferentially integrated into engram circuits, supporting the notion that these attributes promote the rapid recruitment and stabilization of memory-specific assemblies (Ghandour et al., 2019; Tonegawa et al., 2018; Lau et al., 2020).

Engrams are not solely neuronal in nature. Notably, astroglial engrams represent an emerging research area, and related findings are preliminary relative to the well-established literature on neuronal engrams. Recent evidence expands the engram framework to encompass a multicellular model that includes astrocyte-derived functional ensembles, termed astroglial engrams (Sánchez Romero and Navarrete, 2026). Neuronal engrams are conceptually viewed as the core of the memory trace, predominantly involving excitatory glutamatergic neurons, while inhibitory neurons modulate ensemble activity to preserve circuit balance. In contrast, astroglial engrams consist of functional and heterogeneous subsets of astrocytes that are enriched for genes associated with calcium-dependent exocytosis and glutamate release (de Ceglia et al., 2023). These astrocytes can be recruited as sparse ensembles during learning and exhibit coordinated activity with neuronal engrams, thereby forming multicellular memory units. Their contribution is facilitated by bidirectional signaling at the tripartite synapse, which includes gliotransmitter pathways involving D-serine and adenosine, collectively supporting engram formation and stability (Sánchez Romero and Navarrete, 2026; Santello et al., 2019). Reactivation of neuronal engrams can induce calcium responses in associated astrocytic ensembles, and disruption of this neuron-astrocyte coupling impairs complete memory recall (Sánchez Romero and Navarrete, 2026). These findings underscore the functional interdependence between neuronal and astroglial engrams in the process of memory.

**FIGURE 1:**
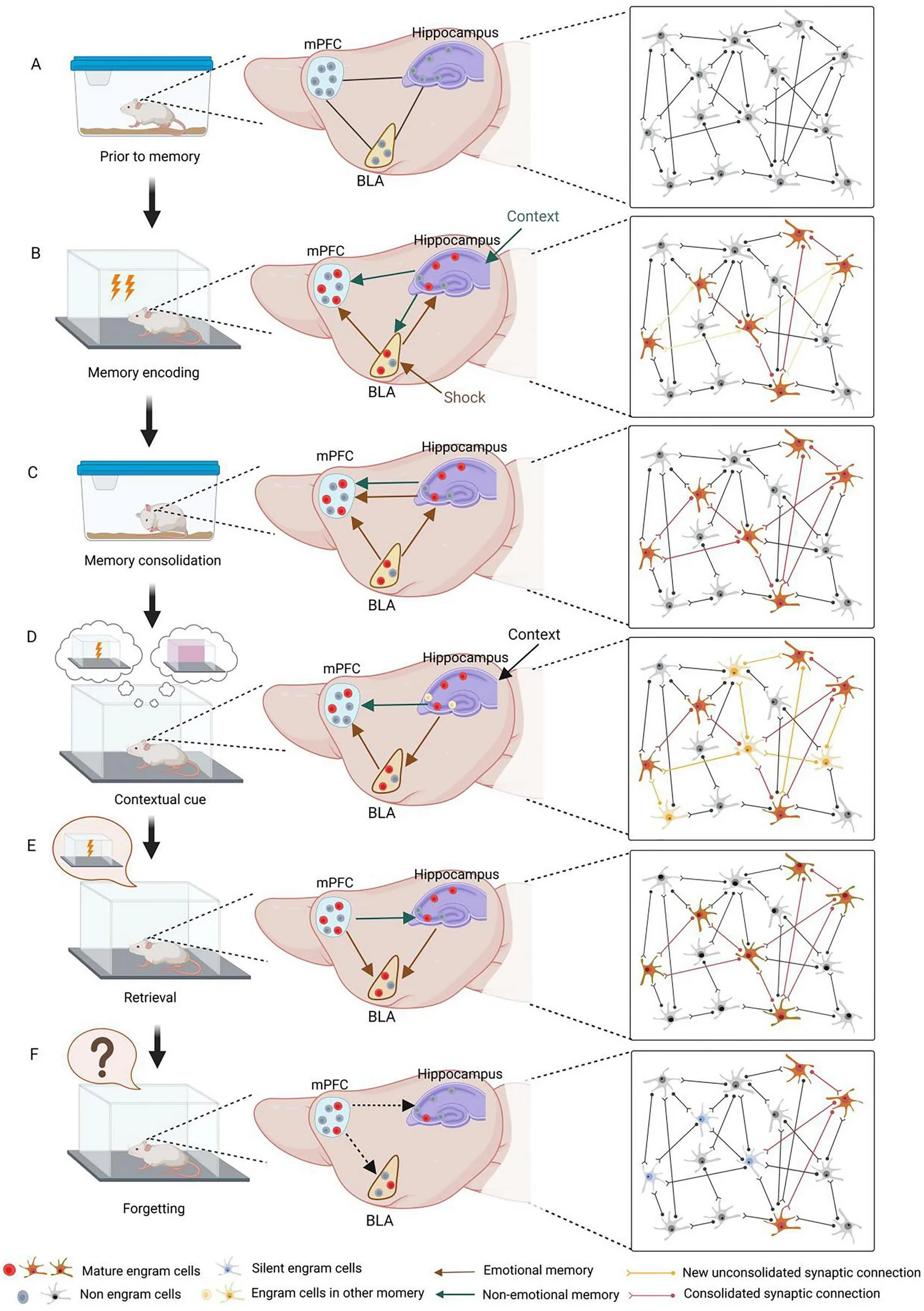
A mouse model of memory formation and forgetting in a representative hippocampus–BLA–mPFC circuit. Schematic illustrating stage-dependent engram dynamics during contextual fear learning, emphasizing coordinated interactions among the hippocampus, BLA, and mPFC. **(A)** Prior to learning, no memory-specific ensembles are recruited and synaptic connectivity remains at baseline. **(B)** During encoding, contextual information and aversive valence jointly recruit sparse neuronal ensembles across regions, initiating new synaptic connections. **(C)** During consolidation, ensemble connectivity is strengthened and stabilized, supporting a mature memory trace. **(D)** A contextual cue triggers partial ensemble activation and begins reinstatement of the distributed engram state. **(E)** During retrieval, coordinated reactivation across the circuit supports successful recall through ensemble reinstatement. **(F)** During forgetting, weakened connectivity and partial silencing of ensemble components prevent full reinstatement, resulting in retrieval failure. This diagram is intended as a conceptual example of distributed engram coordination across memory phases, rather than a universal circuit for all memory types. This figure was created with BioRender.com.

Engrams at the circuit level are dispersed among interconnected brain regions, with their development, reinforcement, recall, and decay being orchestrated in a stage-specific manner involving these regions collaborating, rather than being driven by solitary activity in a singular site (Roy et al., 2022; Miyawaki and Mizuseki, 2022). An illustrative instance is the hippocampus-basolateral amygdala (BLA)-medial prefrontal cortex (mPFC) network, in which contextual representations, emotional significance, and integrative regulatory mechanisms collectively influence the activation and reactivation of memory ensembles (Figure 1). The functional significance, molecular underpinnings, and circuitry dynamics of engram cells spanning critical brain areas are outlined in Table 1.

**TABLE 1:** Summary of brain region–specific engram cell functions in memory processing.

Brain region	Memory stage	Functional role	Molecular mechanisms	Experimental models	Techniques	References
Hippocampus	Encoding consolidation retrieval	Forms episodic details and spatial maps; systems consolidation; rapid recall (recent memory).	CREB-dependent transcription; upregulation of BDNF/Arc; enhanced synaptic LTP	Fear conditioning; spatial tasks	Optogenetics; TRAP; calcium imaging	Hockeimer et al., 2025 Guskjolen and Cembrowski, 2023 Zhang C. L. et al., 2024 Long and Kuhl, 2021 Miranda et al., 2024
Amygdala	Encoding retrieval	Imbues memories with emotional valence (fear/reward); triggers emotion-linked recall.	Stress hormone modulation (NE, Cortisol); GR/CRF activation; MAPK pathway involvement	Tone-shock conditioning	Chemogenetic inhibition (DREADDs); c-Fos tagging	Zhang H. et al., 2024 Qasim et al., 2023 Santos et al., 2024
Medial Prefrontal Cortex (mPFC)	Consolidation, remote retrieval	Stabilizes long-term memories (systems consolidation endpoint); conscious/strategic recall	CREB-dependent consolidation; mTOR pathway regulation of synaptic structure; synaptic remodeling and reconsolidation mechanisms	Delayed match-to -sample; contextual fear conditioning; object-location task	Optogenetic tracing; c-Fos tagging + transcriptomics; calcium imaging	Cassel et al., 2024 Centofante et al., 2024 Delgado-Sallent et al., 2023 Wang et al., 2022
Entorhinal Cortex (EC)	Encoding, retrieval	Preprocesses/integrates spatial/episodic info (hippocampal gateway); aids pattern completion.	LTP; grid cells; Reelin; synaptic tagging	Object-location association; temporal sequence memory	Optogenetics; TRAP; calcium imaging	Sullivan et al., 2024 Zhang et al., 2023
Parietal Cortex (PC)	Encoding, retrieval	Allocates/spatially guides attention; processes spatial relationships	NMDA-dependent LTP; top-down and bottom-up circuit integration	Virtual reality navigation tasks	Calcium imaging; functional imaging (fMRI)	Howard et al., 2024 Das and Menon, 2024
Striatum	Encoding, retrieval	Encodes/executes habits, skills and stimulus-response associations	Dopamine D1R signaling; Akt/GSK-3β pathway; Corticostriatal LTP	Operant learning; reward-based tasks	Multi-electrode recording; DREADDs; behavioral tracking	Farmani et al., 2024 Markman et al., 2024
Anterior Cingulate Cortex (ACC)	Consolidation, retrieval	Emotional-cognitive integration; conflict memory	Glutamate receptor signaling; ACC-amygdala connectivity	Fear learning; social defeat model	Optogenetics; chemogenetics; *in vivo* recording	Kol et al., 2020 Huang et al., 2021
Posterior Cingulate Cortex (PCC)	Retrieval	Episodic memory retrieval; contextual memory	DMN-related synchronization; hippocampal coupling	Contextual fear conditioning; virtual navigation	fMRI, calcium imaging, electrophysiology	Huang et al., 2021 Daviddi et al., 2024
Hypothalamus	Encoding, consolidation	Stress-memory interaction; emotional memory modulation	HPA axis (CRH, cortisol); orexin and vasopressin signaling	Restraint stress, predator odor exposure	Immunohistochemistry, DREADDs, fiber photometry	Liao et al., 2024 Qin et al., 2022 Mutlu-Burnaz et al., 2022

However, it is important to note that the field currently lacks a universally accepted definition of what constitutes an “engram cell.” Most studies rely on immediate early gene (IEG) expression to tag learning-activated neurons, but recent evidence shows that these markers exhibit markedly different co-expression patterns depending on the valence of the experience and brain region (Arai et al., 2025). Moreover, distinct subtypes of inhibitory interneurons display heterogeneous IEG induction responses (Giorgi and Marinelli, 2021). This raises a critical unresolved controversy: whether engram cells defined by different IEG markers represent overlapping or distinct neuronal populations, and whether conclusions about engram vulnerability in neurodegenerative diseases might be marker-dependent. The field has not yet systematically compared how AD, PD, or HD pathology affects c-Fos-tagged versus Arc-tagged engrams, representing a significant knowledge gap.

### 2.2 A vulnerability logic

Engram function depends on coordinated encoding, consolidation, retrieval, and long-term maintenance. As illustrated in Figure 1, these stages rely on strengthened synapses and cross-regional neural coordination across distributed brain regions, such as the hippocampus, BLA, and mPFC. Disruption at any stage, whether at the level of neuronal excitability, synaptic plasticity, or inter-regional coordination, can compromise engram fidelity and lead to memory failure.

Engram cells are preferentially allocated from neurons with higher baseline excitability and stronger synaptic connectivity (Lau et al., 2020; Tonegawa et al., 2018). This allocation bias positions engram cells closer to functional thresholds: elevated excitability supports ensemble synchronization, yet it also increases susceptibility to excitotoxic and oxidative stress. A parallel constraint may apply to astrocytic engrams, whose Ca^2+^-dependent gliotransmission makes them sensitive to disruptions of calcium dynamics (de Ceglia et al., 2023; Santello et al., 2019). A second vulnerability axis arises from the strong synaptic dependence of engram integrity. The function of engrams is rooted in synaptic plasticity, which provides the structural and functional foundation for memory encoding and storage (Sánchez Romero and Navarrete, 2026; Tonegawa et al., 2018). Consequently, disturbances in synaptic integrity or glia-modulated synaptic transmission can undermine engram stability, as even minor synaptic damage can disrupt the cell-to-cell communication essential for maintaining memory traces (Sánchez Romero and Navarrete, 2026). In line with this synaptic dependence, retrieval is particularly susceptible because it relies on the strength of engram-associated synapses and the ability to suppress interference (Lalo and Pankratov, 2024). Contextual cues can reactivate a subset of engram cells and engage the broader ensemble through pattern completion, with successful recall enhanced by effective cue-engram matching (Frankland et al., 2019; Mocle et al., 2024; Tomé et al., 2024). Thus, early retrieval deficits may indicate diminished engram reactivation (functional “silencing”) even in the absence of overt neuronal loss (Mishra et al., 2022).

A third vulnerability lies in network synchronization and neural oscillations (theta 4–12 Hz, gamma 30–100 Hz), which govern engram function (Marks et al., 2021; Roy et al., 2017). Hippocampal theta rhythms are essential for engram encoding and consolidation; their precise temporal coordination supports the formation of coherent memory ensembles. Conversely, dysregulation of theta oscillations—whether as a loss of power, a shift in peak frequency, or an increase in absolute power—can destabilize memory networks (Frankland et al., 2019; Tomé et al., 2022). Importantly, the directionality of theta changes in neurodegenerative diseases is not uniform. In early-stage AD, numerous studies have reported increased absolute theta power during resting-state EEG, particularly over central and temporal regions, and this elevation correlates with lower Mini-Mental State Examination (MMSE) scores and higher cerebrospinal fluid Aβ1-42 levels (Chetty et al., 2024; Geng et al., 2026). Notably, longitudinal EEG data further refine this picture: relative theta power significantly increased over time in amyloid-positive MCI patients, but no significant change was detected in those who had already advanced to dementia, suggesting that the evolution of theta power is shaped by baseline disease severity rather than following a monotonically increasing course (Scheijbeler et al., 2023). Mediation analyses further suggest that central theta activity statistically mediates the relationship between Aβ deposition and cognitive decline (Geng et al., 2026). Thus, while physiological theta oscillations are indispensable for memory, pathological theta slowing—reflected in enhanced absolute or relative theta power—serves as a marker of neural circuit dysfunction, synaptic stress, and impaired engram reinstatement. This distinction is critical: it is not the presence of theta, but its inappropriate increase (especially in the absence of task demand) that signals vulnerability. In tauopathy models, similar increases in slow-wave activity accompany synapse loss and memory deficits, and in PD, altered oscillatory dynamics (e.g., pathological beta oscillations) can disrupt corticostriatal ensemble coordination.

Memory system consolidation entails the gradual transfer of memory representations from the hippocampus to the neocortex, a process that depends on sleep-related oscillations including hippocampal sharp-wave ripples, cortical slow oscillations, and thalamocortical spindles (Tonegawa et al., 2018). Cross-regional engram activation during sleep relies on these physiological rhythms; disturbances to theta coherence or other oscillatory patterns damage engram integrity and block memory consolidation (Miyawaki and Mizuseki, 2022; Tomé et al., 2024; Pignatelli et al., 2019). Therefore, a nuanced view of oscillatory dynamics—distinguishing adaptive rhythms from maladaptive spectral shifts—is essential for understanding engram vulnerability.

Environmental conditions and physiological states modulate engram vulnerability independent of neuronal death. Enriched environments boost plasticity and engram activity, while impoverished settings produce the opposite effect (Mocle et al., 2024; Roozendaal et al., 2006; McEwen et al., 2016; Marzola et al., 2023; Sale et al., 2014; Toda et al., 2019). Stress exerts dose- and time-dependent impacts: acute stress aids memory encoding, while chronic stress impairs plasticity. Social enrichment can alleviate such detrimental outcomes (Han et al., 2022; Lazarov et al., 2024; O’Leary et al., 2024; Li et al., 2020).

The proposed excitability-vulnerability trade-off, while conceptually appealing, is not without contradictory findings. Some studies suggest that moderately increased neuronal excitability can be compensatory rather than deleterious in early disease stages. For example, in certain AD mouse models, hyperexcitability of dentate gyrus granule cells has been reported as an early event that paradoxically correlates with preserved memory function before transitioning to hypoexcitability with disease progression (Busche et al., 2019). Similarly, in PD, dopaminergic denervation leads to increased firing rates in some striatal neuron subpopulations, which may initially maintain network output before subsequent decompensation (Zhai et al., 2023). These observations suggest a “Goldilocks” principle—neither too little nor too much excitability—but the exact thresholds and their disease-stage dependence remain unresolved. Furthermore, whether astrocytic engrams follow a similar trade-off is entirely unknown, as virtually all existing studies focus on neuronal excitability.

Taken together, the three vulnerability axes—excitability-dependent susceptibility, synaptic integrity dependence, and network synchronization—provide a conceptual scaffold for understanding how engram ensembles might fail in neurodegenerative diseases. In the following sections, we examine how distinct pathological drivers (Aβ/tau, α-synuclein with dopaminergic loss, and mHTT) differentially impinge upon these axes, using AD as the best-characterized example (Section 3.1), followed by PD (Section 3.2) and HD (Section 3.3). For each disease, we map the molecular and circuit evidence onto the three vulnerability axes, thereby testing the generalizability of the framework and identifying disease-specific deviations.

### 2.3 Shared amplifiers

Beyond the intrinsic vulnerabilities associated with the excitability-plasticity tradeoff, synaptic dependence, and network coordination, engram dysfunction can be significantly exacerbated by shared pathological amplifiers that operate across various neurodegenerative contexts, rather than through strictly disease-specific pathways. Microglial activation and complement-mediated synaptic remodeling can heighten engram vulnerability by diminishing synaptic connectivity and reducing ensemble fidelity. The microglia-complement axis serves as a common amplifier in AD, PD, and HD (see section 3.4).

Sleep disturbance is another key amplifier that impairs engram consolidation and persistence, and its relevance is underscored by the high prevalence of sleep disorders in AD, PD, and HD. In amnestic AD, disrupted slow-wave sleep (SWS) is associated with impaired memory consolidation, and prior work suggests it may also impair glymphatic clearance of Aβ, but evidence for accelerated tau accumulation or a vicious cycle is lacking in the present studies (Falgàs et al., 2023; Gupta and Soltanian, 2026). In idiopathic rapid eye-movement sleep behavior disorder (iRBD), a prodromal condition of PD, RBD precedes motor symptoms, but the study found no significant correlation between iRBD and reduced striatal DAT binding or cognitive decline over 2 years (Roascio et al., 2022). In HD, sleep fragmentation and circadian dysrhythmia are early features, and mHTT-related mitochondrial dysfunction is well established (Bartman et al., 2024; Saade-Lemus and Videnovic, 2023). However, whether sleep/circadian abnormalities exacerbate these cellular pathologies remains unknown based on the current literature.

Mechanistically, SWS and REM sleep rely on distinct oscillatory patterns to stabilize declarative and procedural memories respectively (Marshall et al., 2020; Rasch and Born, 2013; Yuksel et al., 2025; Cairney et al., 2015). During sleep, reactivation of engrams acts as neural rehearsal to consolidate memories; sleep disorders disrupt this process and accelerate engram decay (Klinzing et al., 2019; Rao-Ruiz et al., 2021). Memory consolidation also requires functional coupling between the hippocampus and mPFC (Takehara-Nishiuchi et al., 2006; Table 1). Thus, sleep disturbance acts not as an isolated factor, but as an interactive amplifier that exacerbates disease-specific pathology and undermines the cross-regional coordination essential for engram persistence.

## 3 Disease-specific engram vulnerability: drivers and mechanisms

Aging establishes a permissive substrate for engram vulnerability by diminishing neurogenesis, weakening synapses, and reducing neuromodulatory support, which consequently heightens susceptibility to neurodegenerative pathology (Lazarov et al., 2024; Lazarov and Hollands, 2016). Since engram activity is highly region-specific and closely associated with distinct memory circuits, the primary brain regions involved in engram processing are summarized in Table 1.

During aging, neurogenesis in the dentate gyrus of the hippocampus declines significantly, which may limit the functional integration of newly generated neurons into the memory engram circuit and potentially impair the structural plasticity necessary for the renewal of the episodic memory network (Kempermann, 2022; Christian et al., 2014). Concurrently, reductions in acetylcholine and dopamine weaken signal transmission and information integration within engram circuits (Nimgampalle et al., 2023; Lee and Kim, 2022; Echeverria et al., 2023). Increased oxidative stress and chronic neuroinflammation further destabilize engram-supporting synapses by impairing mitochondrial function, disrupting membrane integrity, and altering microglia-mediated synaptic remodeling and neurotrophic support (Lazarov et al., 2024; Dash et al., 2025; Liguori et al., 2018; Chakrabarti and Bisaglia, 2023; Singh et al., 2022). Astrocytes may also play a role by reducing transmitter release and weakening synaptic support functions, thereby compromising the synaptic and circuit mechanisms that underpin engram formation and stability (Lalo et al., 2014).

Neurodegenerative disorders build upon this vulnerable baseline by introducing disease-specific factors that preferentially disrupt synaptic programs and circuit interactions that support engrams. It should be noted that mechanistic studies of engram dysfunction are far more comprehensive in AD; evidence from PD and HD is predominantly indirect and derived from broader neural circuit analyses. In the following sections, we summarize the primary pathological drivers and the associated mechanisms of engram vulnerability in AD, PD, and HD. We then integrate common amplifiers that transcend disease boundaries (Table 2).

**TABLE 2:** Impact of neurodegenerative pathologies on engram cell function and molecular mechanisms.

Pathology	Effects on Engram cells	Mechanism	References
Aβ pathology	Disrupts synaptic structure; impedes signal transduction; influence on the recruitment of Engram cells	NMDA receptor overactivation→Ca^2+^ overload, PSD-95 degradation; Inhibition of α7-nAChR function → reduced dopamine release; suppression of glutamate release via mGluR5; inhibition of AHN→impaired recruitment of Engram cells	Yu et al., 2023 Ortiz-Sanz et al., 2022 Talantova et al., 2013 Roberts et al., 2021 Abd-Elrahman et al., 2020 Mishra et al., 2022
Tau pathology	Microtubule depolymerization, impaired axonal transport; disrupted synaptic protein expression and signaling	GSK-3β activation → inhibition of Akt/CREB/BDNF pathway; mTOR signaling dysregulation → impaired synaptic protein synthesis; PSD-95 degradation, synaptic architecture collapse.	Lei et al., 2025 Abuelezz and Hendawy, 2023 Wu B. et al., 2019
Neuroinflammation (Microglial activation)	Excessive synaptic pruning, synaptic signaling imbalance; disrupted network synchrony and stability	Complement C1q/C3-mediated synaptic phagocytosis; disruption of glutamatergic-GABAergic balance; activated Rac1→ATG7→microglia→ TLR2/4→memory destabilization; reduced AHN	York et al., 2021 Wu et al., 2023 Hong et al., 2016 Chen et al., 2024 Wang et al., 2023 Bie et al., 2019 Zaben et al., 2021
Oxidative stress	Cellular enzyme damage, DNA damage; apoptosis and energy metabolism dysfunction	ROS activation of MAPK/NF-κB pathways → mitochondrial dysfunction (loss of membrane potential, cytochrome C release) → caspase-3 activation (apoptosis); Energy metabolism impairment.	Hosseini et al., 2024 Amoroso et al., 2023 Wen et al., 2025
α-Synuclein Aggregation	Inhibits synaptic vesicle fusion and dopamine transmission; causes aberrant synaptic transmission	Aberrant binding of α-Syn to SNARE proteins (e.g., VAMP2, SNAP-25) → impaired vesicle fusion → neurotransmitter release dysfunction.	Gao et al., 2023 Fusco et al., 2016 Sharma and Burré, 2023
TDP-43 Pathology	RNA metabolism dysregulation (splicing, transport, stability); impairs synaptic protein translation and plasticity	TDP-43 loss of function → aberrant splicing/stability of synaptic mRNAs (e.g., UNC13A) → stress granule (SG)/mRNP granule abnormalities → impaired synaptic protein synthesis and function.	Bademosi and Walker, 2022 Dubinski et al., 2023 Horio et al., 2023
mHTT	CREB/CBP signaling dysregulation → decreased BDNF expression; Mitochondrial dysfunction; Glial cell activation	mHTT aberrantly disrupts CREB/CBP interaction → BDNF transcriptional suppression; mTOR/Akt signaling imbalance; induction of neuroinflammation; mitochondrial dysfunction causing energy metabolism disruption.	Yang Y. et al., 2022 Martín-Flores et al., 2020 Jeon et al., 2016 Zhang et al., 2025

### 3.1 Alzheimer’s disease (AD)

#### 3.1.1 Core pathological drivers: Aβ and tau

AD is the most common cause of dementia worldwide, accounting for an estimated 50%–70% of all cases (International Alzheimer’s Disease, 2023a). According to the World Health Organization (WHO), over 55 million people are currently living with dementia globally, with approximately 10 million new cases emerging each year [84]. This number is projected to rise to around 78 million by 2030 and 139 million by 2050 (World Health Organization [WHO], 2023; International Alzheimer’s Disease, 2023b).

The hallmark episodic memory loss in AD is closely linked to early hippocampus-dependent dysfunction and synaptic disconnection, and is primarily associated with two core pathological processes: Aβ pathology and tau pathology (Lazarov et al., 2024; Wu et al., 2018; Marcatti et al., 2022; Busche et al., 2019). Aβ pathology progresses along a continuum, transitioning from soluble species to oligomers and ultimately forming plaques. In contrast, tau becomes abnormally hyperphosphorylated, mislocalizes from axons, and aggregates into neurofibrillary tangles (NFTs). Although Aβ and tau can be characterized as distinct pathological axes, they frequently co-occur and interact throughout disease progression. Together, they contribute to synaptic failure and network disconnection, which are particularly relevant to early memory impairment (Table 2).

#### 3.1.2 Engram vulnerability mechanisms

The general vulnerability axes outlined in section “2.2 Descriptive statistics and correlations”—namely, the excitability-dependent trade-off, synaptic integrity dependence, and network synchronization—are recapitulated and exacerbated by AD-specific pathology. Hippocampal engram dysfunction in AD is a multifaceted process influenced by the combined effects of Aβ oligomers and hyperphosphorylated tau on each of these axes: (i) at the synaptic level, direct disruption of glutamatergic transmission and spine stability; (ii) at the level of neurogenesis-related circuit plasticity, limiting the recruitment and integration of new neurons into engram networks; and (iii) at the level of distributed circuit interactions, degrading long-range coordination between the entorhinal cortex, hippocampus, and posterior cingulate cortex.

Hippocampal engram dysfunction in AD is a multifaceted process influenced by the combined effects of Aβ oligomers and hyperphosphorylated tau on synapses, neurogenesis-related circuit plasticity, and distributed circuit interactions. Notably, engram impairment may occur prior to observable neuronal loss, indicating a functional stage where a memory trace remains but is increasingly difficult to access. In AD mouse models, optogenetic activation of tagged engram cells can restore recall even when natural cues are ineffective, thereby supporting a “silent engram” state that signifies impaired access rather than complete loss of storage (Roy et al., 2016). This state correlates with decreased dendritic spine density, which precedes neuronal death and serves as a structural basis for retrieval impairment (Lazarov et al., 2024). In early AD, the metabolic decline of the posterior cingulate cortex (PCC) is closely linked to the disruption of functional connections between this region and the hippocampus. This disruption may collectively contribute to the functional impairment of the hippocampal memory imprint during the memory retrieval process, ultimately manifesting as episodic memory disorders (Vanneste et al., 2021; Teipel and Grothe, 2016; Poll et al., 2020). Data from the APP/PS1 model further substantiate a transition from “active” to “silent” hippocampal engrams that aligns with spatial memory deficits, indicating retrieval failure rather than loss of stored information (Roy et al., 2016; Perusini et al., 2017). As illustrated in Figure 1, this failure to reinstate engram activity reflects diminished synaptic connectivity and partial silencing of ensemble components, rather than complete erasure of the memory trace.

#### 3.1.3 Synaptic-level engram impairment and excitability-dependent susceptibility

AD primarily affects the hippocampal dentate gyrus (DG), CA1 regions, entorhinal cortex and other cortical memory networks at early disease stages. Compelling optogenetic studies in AD mouse models demonstrate that reactivating hippocampal engrams can rescue memory retrieval, indicating that early memory deficits arise largely from impaired engram excitability, synaptic integrity and reactivation capacity, rather than permanent loss of memory storage. This causal approach builds upon the foundational demonstration that optogenetic activation of tagged engram cells is sufficient to elicit memory recall in healthy animals, and has since been applied to disease contexts (Roy et al., 2016; Perusini et al., 2017; Liu et al., 2012). Consistently, a broad consensus in engram research holds that neuronal intrinsic excitability and CREB activity govern the likelihood of a neuron being recruited into memory ensembles, with highly excitable neurons preferentially allocated to engrams (Josselyn and Frankland, 2018). Accordingly, in AD, engram neurons with high intrinsic excitability are particularly vulnerable to Aβ, tau pathology, disrupted calcium homeostasis, synaptic loss and inflammatory microenvironments, showing excitability-dependent functional impairment.

At the synaptic level, Aβ oligomers and hyperphosphorylated tau disrupt both pre- and postsynaptic processes required for stable coupling among engram cells. Postsynaptically, Aβ oligomers act through NMDARs and AMPARs to disturb calcium homeostasis, activating calcium-dependent enzymes (e.g., calpain) that degrade postsynaptic density components and destabilize synaptic structure and plasticity (Martinez et al., 2024; Prikhodko et al., 2024). Under pathological conditions associated with AD, the abnormal interaction between presynaptic Aβ oligomers, alpha7 nicotinic acetylcholine receptors (α7nAChRs), and metabotropic glutamate receptor 5 (mGluR5) may impair the release dynamics of neurotransmitters and enhance synaptic inhibition, thereby reducing the fidelity required for precise neural transmission among engram cells (Rennie, 2025; Barykin et al., 2019; Wang et al., 2024a). In addition, Aβ oligomers can perturb membrane properties and promote sustained ionic dysregulation that further compromises neuronal viability (Pannaccione et al., 2020; Demuro et al., 2011; Bode et al., 2017).

Beyond these canonical routes, Aβ oligomers have been reported to engage non-canonical signaling modules that contribute to spine elimination and metaplastic changes. For example, mGluR1-dependent, AKAP150-anchored signaling can trigger intracellular calcium release and activate calcineurin, which dephosphorylates and activates cofilin to dismantle actin structure and drive dendritic spine loss (Prikhodko et al., 2024). In related work, Aβ oligomers can inhibit NMDAR-mediated calcium influx-particularly via GluN2B-containing receptors-thereby impairing synaptic plasticity through a mechanism distinct from simple NMDAR overactivation (Zhang et al., 2022; Li et al., 2011; Kessels et al., 2013; Ferreira et al., 2012; Kervern et al., 2012). Metaplastic activation of CaMKII has also been implicated as an additional layer that primes synapses for dysfunction (Opazo et al., 2018). Aβ oligomers further induce pathophysiological mGluR5 signaling in a sex-selective manner (predominantly in males) in AD models and human cortical tissue, a phenomenon linked to exacerbated Aβ pathology and cognitive impairment (Abd-Elrahman et al., 2020). Aβ-driven calcium dysregulation can also be reinforced by impaired calcium regulators such as the Na^+^/Ca^2+^ exchanger, compounding ionic instability and neuronal stress (Pannaccione et al., 2020; Fani et al., 2021).

Tau pathology amplifies synaptic failure through complementary mechanisms. Hyperphosphorylation causes tau to dissociate from microtubules, leading to cytoskeletal instability and impaired axonal transport of synaptic cargo and mitochondria, which weakens energy support and neurotransmission at engram synapses (Colom-Cadena et al., 2023; Tzioras et al., 2023; Wu et al., 2021; Alonso et al., 2018). Tau-mediated transport deficits can further impair mitochondrial delivery to engram synapses, producing local energy shortage and elevating oxidative stress, thereby weakening synaptic function and engram activity (Wu et al., 2021). At the postsynapse, tau can disrupt PSD organization and receptor clustering through interactions involving PSD-95 and NMDAR-associated complexes (Shen et al., 2023; Italia et al., 2022). In addition, tau pathology interferes with plasticity-related signaling needed for consolidation, including CREB (cAMP Response Element-binding Protein)-dependent transcription and mTOR-dependent protein synthesis (Yin et al., 2016; Bloom and Norambuena, 2024; Peng et al., 2023; Yang et al., 2020; Grimes and Jope, 2001; Chatterjee and Beaulieu, 2022; Iqbal, 2024). In AD-relevant signaling cascades, Akt/GSK-3β dysregulation has been linked to reduced CREB phosphorylation and diminished BDNF (Brain-Derived Neurotrophic Factor) expression, while mTOR inhibition compromises the protein synthesis required for engram consolidation (Peng et al., 2023; Grimes and Jope, 2001; Chatterjee and Beaulieu, 2022; Iqbal, 2024). Collectively, these Aβ- and tau-driven synaptic and signaling defects weaken functional coupling among engram cells, degrade ensemble fidelity, and promote the “silent engram” phenotype observed in early stages (Roy et al., 2016). Operational readouts at this level therefore include engram-specific synaptic integrity (e.g., spine density within tagged engram populations) and the efficiency of cue-driven engram reactivation as an index of accessibility.

**FIGURE 2:**
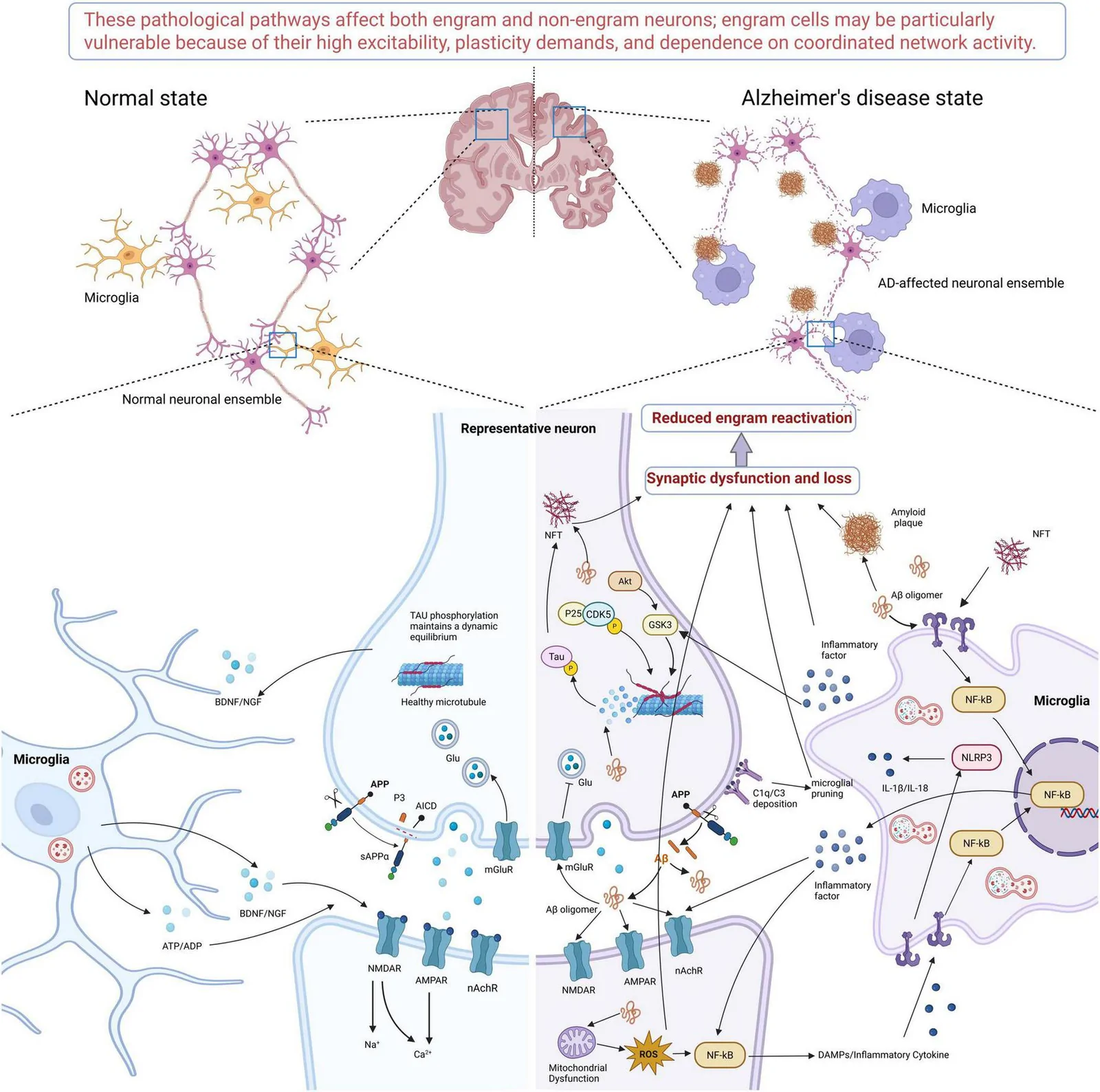
Microglia–neuron interactions and preferential engram vulnerability in Alzheimer’s disease. The left panel illustrates a physiological state in which homeostatic microglia support synaptic maintenance and neuronal connectivity. The right panel summarizes representative pathological processes in AD. These pathways are not specific to engram cells and can also affect non-engram neurons across cortical and subcortical regions. Engram cells may nevertheless show greater functional vulnerability because of their high excitability, plasticity demands, and dependence on coordinated network activity. Aβ oligomers and plaques, together with hyperphosphorylated tau, induce synaptic stress and activate microglial NF-κB and NLRP3 signaling, increasing the release of inflammatory mediators, including IL-1β, TNF-α, and ROS. Complement deposition at synapses, particularly C1q and C3, promotes microglial engulfment and synaptic pruning. In parallel, Aβ interactions with NMDARs, AMPARs, mGluRs, and α7nAChRs disrupt calcium homeostasis and promote mitochondrial dysfunction and oxidative stress. These processes converge on synaptic dysfunction and loss, impaired neuronal communication, and reduced engram reactivation. The molecular components shown are representative and do not constitute an exhaustive pathway map. This figure was created with BioRender.com.

The pathological crosstalk between engram cells and microglia in AD, which amplifies synaptic dysfunction through inflammatory signaling and oxidative stress, is summarized in Figure 2.

#### 3.1.4 Neurogenesis-linked engram impairment

Neurogenesis-related constraints further contribute to hippocampal engram vulnerability by limiting circuit updating and structural plasticity. In familial AD (FAD) mouse models, fewer newborn neurons are recruited into memory engrams, and integrated immature neurons exhibit reduced dendritic spine density and altered transcriptomic profiles, which can restrict the flexibility of engram circuits required for updating and maintaining memory networks (Lazarov et al., 2024; Mishra et al., 2022). In early models of AD, the level of neurogenesis in the dentate gyrus may be similar to that in control groups. However, impaired synaptic integration and circuit connection function of newly generated neurons, rather than a reduction in the quantity of neurogenesis itself, may serve as the major limiting factor for the dysfunction of early memory engrams (Lazarov et al., 2024; Bostancıklıoğlu, 2020). Consistent with this view, progressive spine loss in dentate gyrus engram cells correlates with amnesia and provides a structural basis for retrieval impairment (Choi and Bong-Kiun, 2022; Suratkal et al., 2021). Together, impaired incorporation and maturation of newborn neurons and compromised synaptic integration create a neurogenesis-linked bottleneck that reduces the robustness of hippocampal engram ensembles in AD (Disouky and Lazarov, 2021). Accordingly, actionable readouts include the recruitment rate of adult-born neurons into DG engrams and their maturation/integration status, assessed by their synaptic development and contribution to ensemble reinstatement.

#### 3.1.5 Circuit-level engram impairment

Circuit-level disruptions amplify synaptic and cellular vulnerabilities by degrading information routing and long-range coordination required for engram reinstatement. A core system affected early in AD is the entorhinal-hippocampal circuit, which serves as the primary interface for encoding spatiotemporal and contextual memory (Gómez-Isla et al., 1996). This circuit is functionally organized into two complementary streams: the medial entorhinal cortex (MEC), which conveys spatial information, and the lateral entorhinal cortex (LEC), which carries non-spatial, contextual information (Wang et al., 2018; Masurkar et al., 2017; Tozzi et al., 2024; Igarashi, 2023). Under physiological conditions, the distinct connectivity of MEC and LEC ensures the precise routing of these signals into different hippocampal subregions, supporting the formation of coherent contextual memory engrams (Nilssen et al., 2019; Tozzi et al., 2024). In early AD, however, Aβ/tau pathology disrupts this functional segregation. The LEC, in particular, exhibits heightened vulnerability, leading to degraded contextual signal transmission into the hippocampus (Khan et al., 2014; Save and Sargolini, 2017). Consequently, the integration of spatial and contextual information in the hippocampus is impaired, compromising both the formation and reinstatement of contextual memory engrams by reducing input specificity and disrupting the organized routing of information into hippocampal subregions.

At the network level, PCC hypometabolism-a hallmark of early AD-impairs its modulatory influence on hippocampal engram reactivation and is associated with reduced PCC-hippocampal functional connectivity (Mutlu et al., 2016; Riha et al., 2008). Compromised long-range network coordination and diminished neural integration, as commonly observed in disorders of consciousness, further exacerbate engram disconnection in AD (Wang et al., 2024b). In parallel, tau pathology is associated with reduced functional connectivity of the mPFC within the default-mode network, which may contribute to impaired systems consolidation of remote memories (Guzmán-Vélez et al., 2022; Figure 1). Notably, some early AD mouse models show preserved preferential functional connectivity between EC and DG engram cells, suggesting that gross circuit architecture can remain partly intact even when natural recall fails (Roy et al., 2016). This dissociation supports a model in which early memory impairment reflects degraded ensemble reinstatement and long-range coordination rather than wholesale circuit collapse.

Together, synaptic destabilization (Aβ/tau), neurogenesis-linked integration constraints, and circuit/network-level dyscoordination converge to reduce the accessibility and fidelity of hippocampal engrams, producing early episodic memory impairment characteristic of AD (Lazarov et al., 2024; Roy et al., 2016; Martinez et al., 2024; Perusini et al., 2017). At the circuit level, actionable readouts include impaired EC-hippocampal information routing and reduced long-range network support for engram reinstatement, measurable as weakened functional connectivity during retrieval.

A long-standing controversy in AD research—whether Aβ or tau is the primary driver of early memory impairment—remains unresolved at the engram level. Some studies argue that soluble Aβ oligomers are sufficient to induce synaptic depression and silent engram phenotypes even in the absence of tau pathology, while others demonstrate that tau is necessary for Aβ-induced network dysfunction (Roy et al., 2016; Busche et al., 2019). The field lacks a systematic comparison of how Aβ vs. tau pathology independently affect engram allocation, stability, and retrieval in the same experimental system. Moreover, the interactive effects of Aβ and tau on engram function—whether additive, synergistic, or sequential—are virtually unexplored. This is particularly important because many AD patients present with mixed pathology, and current therapeutic strategies targeting either Aβ or tau have shown only modest clinical benefits, suggesting that engram-based endpoints might help dissect the contribution of each pathology to memory decline.

The pathological crosstalk between engram cells and microglia in AD, which amplifies synaptic dysfunction through inflammatory signaling and complement-dependent pruning, is summarized in Figure 2 and further discussed in section “3.4 Testing for the mediation model.”

### 3.2 Parkinson’s disease (PD)

#### 3.2.1 Core pathological drivers: dopaminergic degeneration and α-synuclein (α-syn)

PD is characterized by progressive loss of dopaminergic neurons in the substantia nigra pars compacta (SNpc) and the accumulation of Lewy pathology composed of aggregated α-syn (Dauer and Przedborski, 2003). Epidemiological data indicate a marked age dependence, with prevalence increasing substantially in older populations (Tysnes and Storstein, 2017). Genetic studies have identified multiple PD-associated loci and pathogenic genes (Yao et al., 2021). Familial PD includes autosomal dominant mutations such as SNCA, which promote α-syn misfolding and aggregation, as well as autosomal recessive mutations (e.g., PARK2, PINK1, DJ1) that impair mitochondrial quality control and proteostasis, thereby increasing dopaminergic vulnerability and promoting α-syn pathology (Polymeropoulos et al., 1997; Mhyre et al., 2012).

These two drivers-dopaminergic degeneration and α-syn pathology-extend beyond the classic nigrostriatal system as disease progresses. Braak staging supports a stereotyped spread of α-syn pathology from brainstem regions to limbic and, in later stages, neocortical structures that participate in memory networks (Braak et al., 2003). In parallel, SNpc degeneration reduces striatal dopamine availability, compromising dopamine-dependent plasticity that is required for habit and motor-memory encoding in striatal ensembles (Hernandez et al., 2019; Zhai et al., 2023; Xu et al., 2018; Lerner, 2018; Surmeier et al., 2023). Dopaminergic dysfunction may also affect reward-related learning and updating, which depend on intact dopamine signaling in limbic circuitry (Zhang et al., 2020).

#### 3.2.2 Engram vulnerability mechanisms

Currently, direct experimental evidence for striatal engram dysfunction in PD is still limited. PD-related motor and cognitive deficits are hypothesized to stem from impaired formation, stabilization and synchronization of striatal engram ensembles, an inference derived from observed synaptic and circuit abnormalities. These putative deficits are likely driven by dopaminergic degeneration and α-synuclein-mediated cellular stress. The striatum acts as a key relay of corticostriatal pathways and governs habit and response memory. Action sequence reorganization for efficient retrieval is severely disrupted in PD (Graybiel, 1998).

Under physiological conditions, motor learning induces highly specific plasticity in the corticostriatal circuit: training selectively strengthens the outputs of M1 motor engram neurons onto striatal spiny projection neurons (SPNs), accompanied by increased formation and stabilization of dendritic spines on these same M1 engram neurons (Hwang et al., 2022). Dopamine is central to this process, as it modulates the excitability and plasticity of striatal medium spiny neurons (MSNs) via D1/D2 receptor signaling, which critically regulates corticostriatal long-term potentiation and depression. Accordingly, dopaminergic denervation deprives striatal ensembles of this key neuromodulatory signal, resulting in the loss of both LTP and LTD and impairing the stabilization of learned motor sequences—a principle originally established through classic studies on striatal synaptic plasticity and subsequently elaborated in the context of PD models (Hernandez et al., 2019; Zhai et al., 2023; Xu et al., 2018; Surmeier et al., 2023; Calabresi et al., 2000). Nevertheless, motor-skill training engages experience-dependent corticostriatal plasticity even under conditions of reduced dopaminergic tone (Farina et al., 2026; Mitchell et al., 2024). Human studies further demonstrate that regular aerobic exercise increases dopamine release and ventral striatum activity, indicating enhanced corticostriatal plasticity in PD patients (Humiñska-Lisowska, 2024; Mitchell et al., 2024). Although the concept of a motor engram has been extensively investigated in the striatum of healthy animals—most recently, *in vivo* two-photon imaging revealed activity-dependent remodeling of corticostriatal axonal boutons during motor learning—it remains unclear how this framework applies in the dopamine-depleted striatum of PD (Sheng et al., 2025a; Cupolillo et al., 2025).

α-syn pathology can further erode striatal engram fidelity through convergent synaptic and cellular mechanisms. At the synaptic level, misfolded α-syn oligomers directly act at presynaptic terminals to inhibit vesicle fusion and reduce dopamine release, thereby weakening the neurotransmission that supports striatal function (Cardinale and Calabrese, 2023). Consistent with this, α-syn aggregates have been linked to reduced evoked corticostriatal glutamate release and loss of corticostriatal synapses (Brzozowski et al., 2025). In parallel, α-syn pathology drives profound mitochondrial dysfunction, including impaired ATP production and increased reactive oxygen species (ROS) generation; this mitochondrial and oxidative stress is directly linked to destabilized firing patterns (slowed and more irregular firing) in vulnerable neuron populations such as SNc dopaminergic and PPN cholinergic neurons (Geibl et al., 2024). At the microcircuit level, recent *in vivo* two-photon imaging during motor learning has revealed dynamic remodeling of corticostriatal axonal boutons, including activity-dependent formation and elimination that correlate with reward-related movement selectivity and are stabilized by learning (Sheng et al., 2025a,b).

Genetic defects in PD-related genes such as PARK2, PINK1, and DJ1 exacerbate these processes by impairing mitochondrial quality control and proteostatic clearance pathways, creating a cellular environment that favors α-syn accumulation and sustained oxidative stress (Mhyre et al., 2012). Beyond direct synaptic and mitochondrial toxicity, neuroinflammation amplifies this vulnerability through microglial activation and cytokine-mediated synaptic loss, as demonstrated in rodent models of neuroinflammation where hippocampal dendritic spine density, synaptic transmission, and long-term potentiation are impaired (Liaury et al., 2014; Wu et al., 2023). Compounding these effects, astrocytic dysfunction, including impaired SNARE-dependent gliotransmitter release and disrupted calcium-to-release coupling, weakens neuron–astrocyte communication and compromises hippocampal synaptic plasticity, a process critical for memory (Abreu et al., 2023; Pillai and Nadkarni, 2022).

At the circuit level, pathology-driven synaptic dysfunction is accompanied by widespread large-scale network reorganization. In mouse models, α-syn pathology can be seeded in the prefrontal cortex and subsequently spread to other cortical and subcortical regions, including medial temporal structures that support memory and cognitive function (Braak et al., 2003; Weber et al., 2023). Beyond canonical pathological β oscillations, multiple forms of phase-amplitude coupling (including Delta-Beta and Theta-Gamma coupling) are also disrupted and closely linked to motor dysfunction and intervention-related neuromodulation in PD (Kazemi et al., 2025). In PD, multiple forms of phase-amplitude coupling — including delta-beta and theta-gamma — are disrupted and closely linked to reduced motor vigor, and their relationship to motor output is modulated by dopaminergic medication and galvanic vestibular stimulation (Kazemi et al., 2025). In addition to basal ganglia circuit dysfunction, large-scale network disorganization, particularly reduced connectivity between the hippocampi and within the default mode network, is associated with multi-domain cognitive impairments in PD, including visuospatial dysfunction (Zarifkar et al., 2021; Mijalkov et al., 2022). Together, the spread of α-syn pathology to corticolimbic networks, disruption of multiple phase-amplitude couplings, and large-scale functional disconnection converge to impair striatal and cognitive circuits, ultimately giving rise to both motor and non-motor manifestations in PD. Beyond direct synaptic and mitochondrial toxicity, neuroinflammation amplifies this vulnerability through microglial activation and complement-linked synaptic remodeling, as discussed in section 3.4.

### 3.3 Huntington’s disease (HD)

#### 3.3.1 Core pathological drivers: mutant huntingtin (mHTT) and downstream synaptic/transcriptional effects

HD is an autosomal dominant neurodegenerative disorder caused by CAG repeat expansion (typically ≥ 40) in the HTT gene, with core clinical manifestations including chorea, cognitive decline, and neuropsychiatric symptoms (McColgan and Tabrizi, 2018; Pringsheim et al., 2012). Epidemiological studies report substantial geographic variation in prevalence, which is higher in populations of European ancestry than in East Asia, and typical clinical onset occurs between 30 and 50 years of age (Pringsheim et al., 2012; Medina et al., 2022; Ross et al., 2014).

At the pathological level, mHTT exerts toxicity through both nuclear and cytoplasmic mechanisms, leading to early dysfunction and later degeneration within striatocortical systems that are critical for procedural learning, habit formation, and cognitive flexibility. A defining cellular driver is the selective vulnerability of striatal GABAergic MSNs, which constitute the principal neuronal substrate for striatal engram ensembles and show early subtype-specific susceptibility during HD pathogenesis (Lee et al., 2020).

#### 3.3.2 Engram vulnerability mechanisms

Conclusions regarding engram impairment in HD are mainly drawn from indirect circuit observations. In this view, HD-related engram dysfunction can be understood as striatocortical ensemble failure driven by three interconnected pathological cascades: (i) loss of neurotrophic support that abolishes corticostriatal plasticity essential for engram stabilization, (ii) mHTT-mediated transcriptional and vesicular trafficking dysregulation that destabilizes synaptic programs, and (iii) circuit-level desynchronization that undermines ensemble coordination.

First, at the molecular level, a foundational mechanism underlying all three cascades is the disruption of local BDNF-TrkB signaling, which is indispensable for sustaining synaptic plasticity (including LTP) and the local protein synthesis required for stable memory consolidation (Johnstone and Mobley, 2020; Wang et al., 2024d). In HD, mHTT inflicts a dual hit on this pathway. On one hand, it impairs BDNF production in cortical neurons via epigenetic and transcriptional repression, including upregulation of Twist 1, sharply reducing BDNF expression in these neurons (Pan et al., 2018). On the other hand, while wild-type huntingtin enables microtubule-dependent BDNF vesicle transport through the HAP1/p150Glued complex, mHTT binds abnormally and blocks this machinery, reducing the attachment of TrkB-containing vesicles to microtubules and impairing their retrograde trafficking in striatal dendrites (Liot et al., 2013; Gauthier et al., 2004). As a result, BDNF-induced TrkB signaling is severely blunted, as evidenced by decreased ERK phosphorylation and c-fos induction (Liot et al., 2013). Consequently, this combined deficit in anterograde BDNF delivery and retrograde TrkB signaling collapses trophic support for striatal neurons (Baydyuk and Xu, 2014; Table 2).

Second, in parallel to BDNF-TrkB collapse, mHTT drives global nuclear transcriptional dysregulation. Specifically, cleaved N-terminal fragments accumulate in the nucleus, where mHTT sequesters transcription factors (Sp1, NF-Y) and disrupts the basal transcription machinery (Kumar et al., 2014; Yamanaka et al., 2008). This leads to BDNF suppression via REST/NRSF activation (Kumar et al., 2014). Moreover, cytoplasmic mHTT binds mature SREBP and importin β, blocking SREBP nuclear import and downregulating its target genes (Di Pardo et al., 2020).

Third, moving to the circuit level, these progressive molecular and synaptic defects converge to destabilize striatal ensemble dynamics. Consistent with this, in HD mouse models, striatal neurons exhibit increasingly aberrant and uncoordinated firing, accompanied by profound losses of behavioral flexibility (Hong et al., 2012). Extending these findings to humans, in premotor HD, egocentric working memory, which is supported by the dorsolateral caudate, is disproportionately impaired, whereas allocentric working memory, which is supported by the posterior hippocampus, remains relatively spared. Thus, these results suggest early dysfunction of striatal-dependent spatial processing before widespread network failure (Possin et al., 2017).

Finally, as an additional but interacting mechanism, in HD, mHTT-associated cellular stress is accompanied by early microglial and complement activation (see section 3.4). This process can further destabilize ensembles supporting procedural learning and interact with impaired BDNF/TrkB-dependent plasticity.

### 3.4 Shared driver: microglia/complement-linked synaptic remodeling

Across AD, PD, and HD, distinct upstream triggers (Aβ/tau, α-syn with dopaminergic dysfunction, and mHTT) can converge on a common inflammatory-synaptic pathway that weakens engram connectivity. Disease-associated proteins and cellular stress signals engage microglial receptors, including Toll-like receptors (TLRs) and scavenger receptors, and drive a pro-inflammatory program that increases cytokine signaling and oxidative mediators (Calvo-Rodriguez et al., 2020; Ioghen et al., 2021). Key effectors include inflammasome-linked signaling (e.g., NLRP3/IL-1β), TNF-α, and ROS, which together reduce synaptic plasticity capacity and destabilize network activity.

A major link between microglial activation and engram failure is complement-dependent synaptic tagging and pruning. Complement proteins such as C1q and C3 can deposit at synapses and promote microglial phagocytosis, leading to loss of engram-relevant synaptic connections (Wang et al., 2020). This microglia-complement axis therefore provides a shared mechanism by which diverse pathologies translate into synaptic disconnection, reduced ensemble fidelity, and impaired cue-driven engram reactivation.

The concept that complement components such as C1q can deposit at synapses and tag them for microglial phagocytosis was originally established in the context of developmental circuit refinement (Stevens et al., 2007). In AD, Aβ oligomers and hyperphosphorylated tau activate microglia and increase complement components such as C1q/C3, which label hippocampal engram synapses for pruning (Wang et al., 2020; Hong et al., 2016). This synaptic loss can precede overt neuronal death and is associated with reduced engram reactivation during retrieval, consistent with a “silent engram” phenotype. In AD, this complement-microglia axis has been causally linked to engram failure through multiple *in vivo* intervention studies (e.g., C1q or C3 knockout rescues synaptic density and memory recall). In parallel, inflammatory mediators (e.g., IL-1β, TNF-α) can further limit hippocampal plasticity, compounding the loss of ensemble fidelity (Figure 2).

In PD, α-syn pathology together with dopaminergic degeneration shifts microglial states and is associated with complement pathway activation and synaptic pruning in corticostriatal circuits (Zhu et al., 2022; Liu et al., 2022; Chi et al., 2025; Martin et al., 2024). However, direct causal evidence that complement-dependent synaptic tagging and microglial phagocytosis drive engram weakening in PD remains limited. Microglia-derived inflammatory signals and complement-related synaptic vulnerability can weaken corticostriatal synapses and make dopamine-dependent plasticity harder to maintain, thereby reducing the stability of striatal ensembles. At the circuit level, this immune-synapse stress can interact with network dysrhythmia and further degrade ensemble synchronization during motor learning and expression.

In HD, mHTT-associated cellular stress is accompanied by early microglia and complement activation, with increased complement proteins reported around striatal synapses (Kouser et al., 2015; Wilton et al., 2023). Although direct evidence remains less extensive than in AD, studies in patient tissue and HD mouse models have shown that C1q is upregulated and binds corticostriatal synapses, coinciding with reduced PSD-95 and decreased synapse density, consistent with complement-mediated synaptic elimination in striatocortical loops (Wilton et al., 2023). Notably, these findings predominantly derive from pre-symptomatic or early-stage mouse models (e.g., zQ175 knock-in mice), and whether complement pruning directly destabilizes procedural learning ensembles in manifest HD requires further functional interrogation. This process can destabilize ensembles supporting procedural learning and can interact with impaired BDNF/TrkB-dependent plasticity described in HD, further reducing ensemble stability.

Importantly, the differential strength of evidence across diseases may reflect genuine biological differences or simply differences in research attention. In AD, multiple genetic and pharmacological intervention studies have established a causal role for complement-dependent pruning in engram dysfunction. For PD, several studies report elevated complement transcripts (C1q, C3) in post-mortem striatum and in α-synuclein models, but functional intervention studies—such as C1q knockout or C3a receptor blockade—have not yet been published. One unresolved controversy is whether complement activation in PD is a primary driver of synapse loss or a secondary consequence of dopaminergic degeneration. The observation that L-DOPA treatment can modulate microglial activation suggests a link between dopamine signaling and complement pathways, but causality remains unclear. For HD, the elegant study by Wilton et al., provides strong histological evidence for complement deposition at corticostriatal synapses, but it remains unknown whether complement inhibition can rescue procedural learning deficits in HD models—a critical test that has not yet been reported (Wilton et al., 2023).

An important unresolved controversy concerns the dual role of complement in memory. Under physiological conditions, complement-mediated synaptic pruning is essential for developmental circuit refinement and may even contribute to normal forgetting (Wang et al., 2020). In adult hippocampus, tonic C1q expression and microglial pruning are required for the turnover of less-used synapses and for memory updating. Thus, complete inhibition of complement signaling—while protective in AD models—might interfere with normal memory dynamics and cognitive flexibility. Indeed, C1q knockout mice show altered fear extinction learning, suggesting that complement contributes to both forgetting and adaptive memory updating. This creates a therapeutic tension: how to selectively block pathological over-pruning without disrupting physiological pruning? The field currently lacks complement inhibitors that discriminate between these contexts, representing a major translational knowledge gap.

Importantly, the neuroprotective effects of complement inhibition are not uniform across sexes. In the TauP301S tauopathy model, C3 deficiency rescued brain atrophy and synapse loss in male mice but failed to protect females (Wu T. et al., 2019). This sexual dimorphism, likely mediated by estrogenic regulation of microglia and complement expression, highlights a major knowledge gap in current engram-centered frameworks: whether complement-targeting interventions would benefit both sexes equally in AD. Future studies must stratify by sex to avoid overgeneralized conclusions and to guide precision therapeutic strategies.

Sex-dependent differences in complement signaling are emerging in PD, although direct functional evidence remains limited. Cross-sectional studies have reported that in male PD patients, serum C3 levels may be lower than in healthy male controls and female PD patients (Sun et al., 2019). In female PD patients, plasma C3 levels correlate positively with the severity of non-motor symptoms, particularly anxiety and depression (Khosousi et al., 2023). However, evidence for a direct role of C1q in sexually dimorphic microglial activation or for a “microglia-complement axis as a shared amplifier of engram vulnerability” is not provided by these studies and would require additional experimental data, such as from human iPSC-derived microglia models. Future investigations of complement-targeted therapies in PD should consider sex stratification as a relevant design variable.

The mechanism(s) underlying the sex-specific efficacy of mGluR5 negative allosteric modulators in zQ175 HD mice remain unclear, although these compounds reduce mutant huntingtin aggregation and microgliosis in both sexes (Li et al., 2022). In addition to mGluR5-based observations, more direct evidence for sex-dependent complement biology in HD is still scarce. The study by Wilton demonstrated that complement proteins C1q and C3 are upregulated at corticostriatal synapses in HD mice and patients, but whether this complement-mediated synaptic pruning differs between males and females has not yet been analyzed (Wilton et al., 2023). Given that C3 deficiency rescued neurodegeneration in male but not female TauP301S tauopathy mice, it is plausible that complement-targeting strategies for HD may also yield sex-dependent outcomes (Wu T. et al., 2019). Therefore, future preclinical studies in HD models must explicitly re-analyze existing complement data by sex and include sex stratification as a fundamental design principle.

It is important to maintain a balanced perspective here: although engram cells exhibit heightened vulnerability to complement-mediated synaptic loss, as illustrated in Figure 2, this microglia-complement pathological cascade is not exclusive to memory trace ensembles (Wang et al., 2020). Instead, nearly all neuronal populations across vulnerable cortical and subcortical circuits undergo comparable inflammatory synaptic remodeling under neurodegenerative stress, as demonstrated by α-synuclein-induced microglial activation in PD and mutant huntingtin-triggered C1q deposition in HD (Zhu et al., 2022; Wilton et al., 2023). These pathological stimuli drive widespread C1q deposition and microglial phagocytosis at synapses of non-engram neurons, leading to global circuit-level synaptic impairment rather than restricted engram-specific damage. Across diseases, these shared mechanisms converge on synaptic weakening, ensemble desynchronization, and reduced engram reinstatement. The multifaceted impacts of core neurodegenerative pathologies on engram function and their underlying molecular mechanisms are summarized in Table 2.

## 4 An engram-centered therapeutic roadmap for neurodegenerative diseases

### 4.1 Therapeutic framework and modality mapping

An engram-centered therapeutic roadmap prioritizes early suppression of disease-causing pathology, preservation of engram-supporting synaptic plasticity, and recovery of coordinated network activity to enable normal ensemble reactivation. Since engram impairment emerges before substantial neuronal death, early intervention targeting synapses and circuit dynamics holds the greatest therapeutic potential; Table 1 summarizes regional engram distributions and functions, while Table 2, outlines pathological impacts on engrams to guide early therapeutic design. Importantly, none of the pharmacological, genetic or neuromodulatory strategies discussed below exclusively manipulate engram cells—they exert broad effects across excitatory/inhibitory neurons, astrocytes and microglia, and cognitive improvements arise from combined homeostatic regulation of the overall neural microenvironment plus targeted engram protection, rather than engram rescue alone.

**FIGURE 3:**
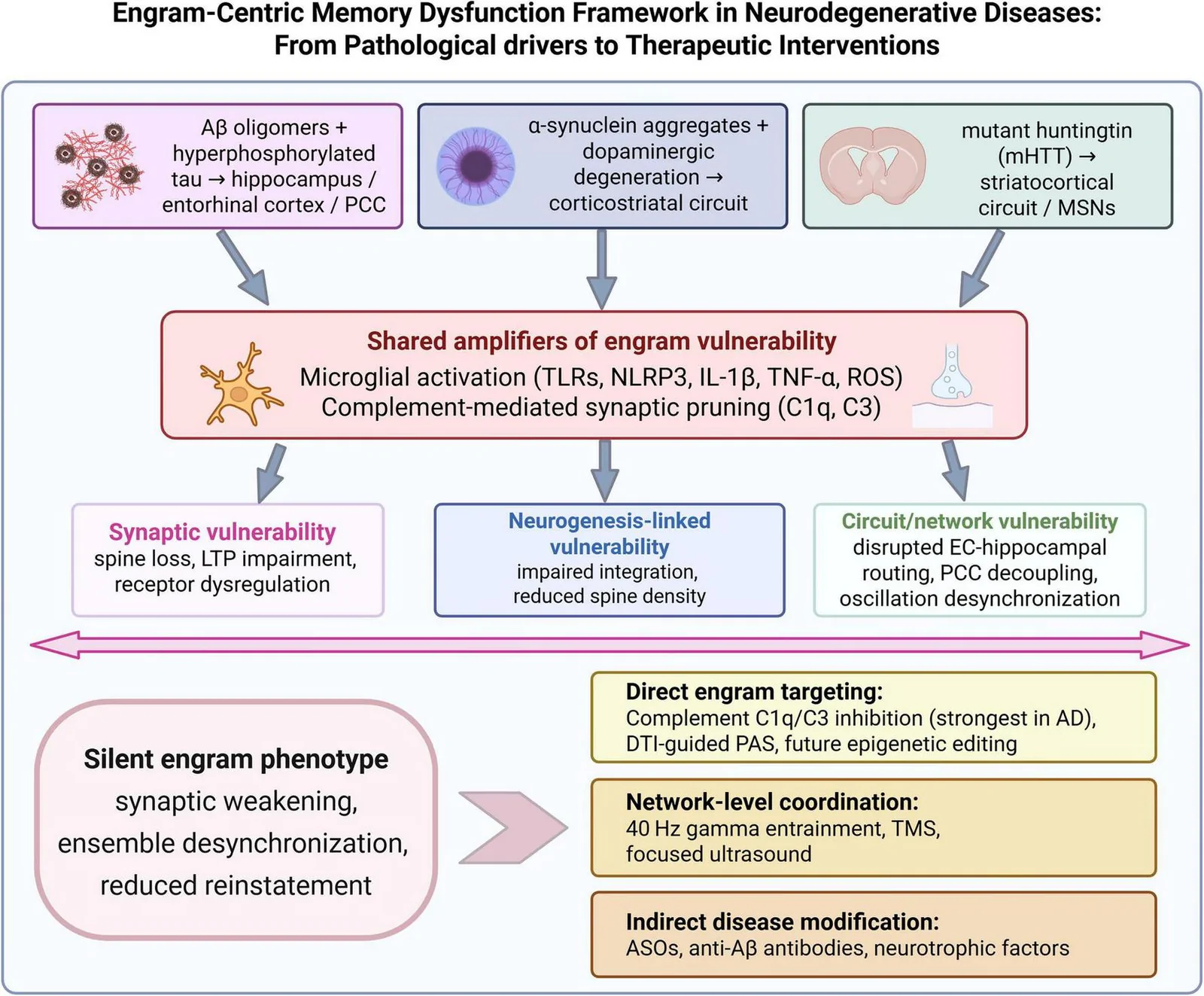
Integrated framework linking disease-specific drivers, shared amplifiers, engram vulnerability, and therapeutic strategies across AD, PD, and HD. Disease-specific drivers, including Aβ/tau in AD, α-synuclein with dopaminergic loss in PD, and mHTT in HD, converge with shared amplifiers such as microglial activation, complement-associated synaptic pruning, and sleep disruption. These processes impair synaptic plasticity and circuit coordination, reducing ensemble stability and reactivation. A silent-engram state is best supported in AD, whereas related ensemble dysfunction in PD and HD remains less directly established. Therapeutic approaches are grouped into pathology reduction, protection of synaptic and ensemble function, and circuit restoration. The illustrated pathways and interventions affect both engram and non-engram neuronal and glial populations. Engram ensembles may nevertheless show greater functional vulnerability because of their high excitability, plasticity demands, and dependence on coordinated network activity. This figure was created with BioRender.com.

We divide engram-protective interventions into two mechanistic tiers: Layer 1 refers to direct engram-targeted therapies that modulate molecular or circuit machinery governing engram formation, storage and retrieval, such as inhibiting C1q/C3 complement pruning, optogenetic/non-invasive neuromodulation to strengthen engram synapses, and prospective epigenetic editing to lock engram cell identity. It should be noted that these tools still act on widespread non-engram neuronal and glial populations and do not selectively modify only memory ensembles. Layer 2 consists of disease-modifying treatments that indirectly safeguard engrams by lowering upstream toxic protein load (Aβ, α-syn, mHTT) or supplying universal neurotrophic support; their engram benefits are secondary outcomes of global neuroprotection and must be verified with engram-specific readouts instead of presumed. Subsequent sections detail interventions categorized by this two-layer system, and Figure 3. provides a visual summary linking disease triggers, shared pathological amplifiers, engram vulnerability pathways and corresponding therapeutic targets.

It is critical to clarify that all therapeutic strategies discussed below are not limited to specific manipulation of engram cells only. Instead, these pharmacological, genetic, and neuromodulatory approaches produce broad biological effects across the full spectrum of central nervous cell populations, including non-engram excitatory/inhibitory neurons, astrocytes, and microglia. Improvements in neurodegenerative pathology are not mediated solely via rescuing engram ensemble function; rather, therapeutic benefits emerge from a multifactorial dynamic regulatory network covering global neural microenvironment homeostasis and engram-specific protection simultaneously.

### 4.2 Direct engram-targeting interventions: from causal proof-of-concept to human translation

#### 4.2.1 Strengthening silent engram synapses: optogenetic evidence

In early AD, memory loss stems from impaired retrieval rather than erasure of memory traces. Shrestha and Klann showed that optogenetic strengthening of hippocampal engram synapses rescues contextual fear memory in early-stage AD mice, confirming that engram cells remain structurally intact despite significant pathology, and that their reactivation directly restores memory (Shrestha and Klann, 2016). This finding fundamentally shifts the therapeutic goal from passive protein clearance to active functional circuit repair. Optogenetics offers high spatiotemporal control and has established causal links between engram manipulation and behavior in animal models, but clinical translation is limited by invasiveness and delivery constraints (He et al., 2025; Yang Q. et al., 2022; Zhang Q. et al., 2024; Yizhar et al., 2011; Goshen, 2014; Adamczyk and Zawadzki, 2020).

It should not be overlooked that optogenetic stimulation also alters general excitability and synaptic transmission of non-engram neurons within the targeted brain region; cognitive improvements cannot be fully explained by selective engram activation alone, reflecting a multifactorial circuit-wide modulation effect (Liu et al., 2023; Watkins de Jong et al., 2023; Mocle et al., 2024).

#### 4.2.2 Complement inhibition: preventing selective engram synapse pruning

The role of complement-linked synaptic pruning offers a mechanistic justification for direct engram protection. However, complement inhibition does not exclusively protect engram synapses. C1q/C3 blockade broadly suppresses pathological synaptic pruning across all neuronal subpopulations and regulates microglial reactivity throughout the brain parenchyma (Yousefpour et al., 2025; Zhong et al., 2023; Xie et al., 2026; Tenner and Petrisko, 2025). The overall neuroprotective phenotype arises from coordinated protection of global synaptic networks and engram ensembles, rather than a sole engram-centered effect (Tenner and Petrisko, 2025; Deivasigamani et al., 2023). In AD, complement C1q can intensify synaptic damage during later pathological stages through classical pathway activation and plaque-associated glial inflammation. In transgenic models of AD, genetic knockout of C1q or inhibition of C1q-related inflammatory pathways preserves synaptic integrity even after formation of fibrillar Aβ plaques (Fonseca et al., 2004). This qualifies as a direct engram-targeting intervention because it specifically interrupts the molecular machinery that tags and eliminates engram-relevant synapses. In PD and HD, equivalent direct causal evidence is currently lacking; complement activation has been reported correlatively, but functional interventions have not yet been tested for their ability to rescue ensemble stability (Zhu et al., 2022; Liu et al., 2022; Wilton et al., 2023) (see section “4.4 Engram-relevant disease-modifying interventions (indirect)” for evidence levels).

Despite the promise of complement inhibition as a direct engram-targeting strategy, several unresolved questions remain. First, the optimal timing of C1q/C3 blockade is unclear. In AD models, complement inhibition is protective when administered early, but its efficacy in later stages with established plaque pathology is less certain (Fonseca et al., 2004). Second, complement proteins have homeostatic functions beyond pruning, including roles in synaptic maintenance and neuroprotection; whether chronic C1q inhibition leads to unexpected adverse effects (e.g., impaired clearance of apoptotic cells or increased susceptibility to infection) has not been thoroughly evaluated in aging brains. Third, it is unknown whether complement inhibition would be equally effective in PD and HD given the different cellular contexts; the absence of functional intervention studies in these diseases constitutes a major knowledge gap.

#### 4.2.3 Non-invasive human translation: targeted circuit neuromodulation

In humans, non-invasive approximations of engram-directed neuromodulation are emerging. Ma et al. used diffusion tensor imaging (DTI)-guided paired associative stimulation (PAS) to precisely target the cortical-hippocampal circuit in amnestic mild cognitive impairment (aMCI) patients. This intervention significantly improved delayed recall, with effects mediated by enhanced hippocampus-precuneus functional connectivity (Ma et al., 2024). Although not yet at single-ensemble resolution, this approach represents a direct engram-relevant intervention because it specifically strengthens the connectivity of a defined memory circuit.

Still, neuromodulation modulates overall neuronal oscillatory activity and synaptic transmission across the stimulated cortical area, acting on both engram and non-engram neurons synchronously (Tan et al., 2020; Shu and Jackson, 2025; Ghandour et al., 2025). Memory recovery relies on multifactorial circuit reorganization instead of exclusive engram modulation (Ghandour et al., 2025; Tomé et al., 2022).

Other non-invasive or less invasive neuromodulation approaches—such as transcranial focused ultrasound, magnetic field modulation, and personalized multi-target TMS—may provide additional routes to modulate memory circuits in humans (Alipour et al., 2025; Zhantleuova et al., 2025). These modalities can be adjusted to restore coordination within specific memory circuits, with efficacy evaluated using engram-relevant endpoints.

#### 4.2.4 Epigenetic stabilization of engram persistence

Memory engram persistence further relies on epigenetic regulation within engram cells. Fuentes-Ramos, Alaiz-Noya, and Barco review that stable epigenetic alterations may encode enduring memories by sustaining gene expression programs. While key modifications remain unknown, next-generation sequencing can profile engram transcriptomes/epigenomes, and CRISPR-dCas9-based epigenetic editing offers a tool to test their causal roles (Fuentes-Ramos et al., 2021). Therapeutically, this opens a future class of direct engram-stabilizing interventions that reinforce engram cell identity without altering synaptic connectivity *per se*. This remains a preclinical strategy but represents a distinct layer from synaptic protection or network neuromodulation.

### 4.3 Network-level coordination: non-invasive neuromodulation for ensemble reinstatement

Network-wide neuromodulation generates global rhythmic changes across widespread neuronal populations rather than selectively tuning engram ensembles alone. The non-invasive 40 Hz gamma entrainment framework was established by a series of seminal studies from Li-Huei Tsai’s MIT Picower Institute group. This line of work first demonstrated that sensory stimulation at 40 Hz—initially through visual flicker, subsequently via auditory tones and combined audiovisual paradigms —rescues oscillatory dysfunction, alleviates Aβ/tau pathology, modulates microglial responses, and restores memory ensemble synchrony in AD models (Iaccarino et al., 2016; Martorell et al., 2019; Adaikkan et al., 2019). Later mechanistic studies further revealed that this entrainment facilitates glymphatic clearance of amyloid via VIP interneuron-mediated regulation of arterial pulsatility, and early clinical evidence suggests that long-term 40 Hz audiovisual stimulation may slow cognitive decline and reduce plasma pTau217 in mild AD patients (Murdock et al., 2024; Chan et al., 2025). Collectively, this body of work provides the mechanistic foundation for the circuit therapeutic framework illustrated in Figure 3. At the macro-network level, coordinating distributed circuits to enhance ensemble synchrony offers a clinically scalable route to engram reinstatement. For example, Lahijanian, Aghajan, and Vahabi found that 40 Hz auditory gamma entrainment restores default mode network (DMN) functional connectivity in dementia patients by synchronizing rhythmic energy across affected regions (Lahijanian et al., 2024). This effect is indirect but engram-relevant because DMN integrity correlates with episodic memory retrieval, and gamma entrainment may facilitate the temporal coordination required for engram reactivation without targeting specific synapses.

Additionally, dynamic engram consolidation highlights the neocortex in remote memory storage. Lee et al. demonstrated that contextual fear memories mature from hippocampal dependence to neocortical stabilization; excitatory connections between prefrontal engram neurons progressively strengthen, providing a synaptic basis for persistent remote memory (Lee et al., 2023). This finding implies that in later disease stages, or for well-established memories, interventions may need to target neocortical engram ensembles (e.g., via repetitive TMS to medial prefrontal cortex), although direct evidence in neurodegenerative diseases is currently lacking.

While non-invasive neuromodulation approaches such as 40 Hz gamma entrainment and TMS show promise, their precise mechanisms of action on engram function remain controversial. Some studies attribute the effects to direct entrainment of neuronal oscillations, while others suggest indirect effects via reduced inflammation or enhanced glymphatic clearance. Moreover, the specificity of these interventions for engram ensembles—rather than global brain activity—is unclear. It remains possible that the observed cognitive improvements are mediated by non-specific arousal or attentional effects rather than genuine restoration of engram reinstatement. Future studies should incorporate engram-specific readouts (e.g., task-related fMRI pattern reinstatement) to distinguish these possibilities.

### 4.4 Engram-relevant disease-modifying interventions (indirect)

#### 4.4.1 Alzheimer’s disease

Pathology-lowering therapies targeting Aβ and tau exert broad cytoprotective effects on all neuronal and glial populations throughout the brain, not only memory engram cells (Chen et al., 2026; Feng et al., 2024; Wen et al., 2026). The alleviation of cognitive dysfunction stems from comprehensive relief of pan-cellular synaptotoxicity, a multifactorial dynamic process where engram rescue is only one component (Feng et al., 2024; Latina et al., 2025; Behroozi et al., 2026). In AD, early network alterations are evident as diminished functional connectivity between the hippocampus and the PCC, as well as other memory-related regions, observed through fMRI. This finding aligns with an initial disruption of hippocampal-PCC coupling and a decrease in the accessibility of hippocampus-dependent memory traces (Wang et al., 2024c; Papma et al., 2017; Sperling et al., 2010; Lombardi et al., 2020; Feng et al., 2019). During this phase, the attenuation of upstream pathology seeks to reduce Aβ/tau-related synaptotoxic pressure, thereby lessening the likelihood of deterioration in hippocampal engram connectivity. These are indirect interventions because they do not directly strengthen engram synapses or block pruning; their effect on engrams depends on early timing to preserve the substrate that engrams require. The role of complement-linked synaptic pruning offers a mechanistic justification for prioritizing early synaptic protection: complement C1q can intensify synaptic damage during later pathological stages through classical pathway activation and plaque-associated glial inflammation. In transgenic models of AD, the genetic knockout of C1q or the inhibition of C1q-related inflammatory pathways has been shown to preserve synaptic integrity, even after the formation of fibrillar Aβ plaques (Figure 2).

#### 4.4.2 Parkinson’s disease

Treatments lowering α-syn burden and replenishing dopamine signaling act across all striatal and cortical neurons, not merely striatal motor engram ensembles. Motor and cognitive improvements arise from multi-cell circuit restoration rather than exclusive engram protection. In PD, early deficits in motor learning are closely associated with impaired striatal plasticity, and α-syn pathology has been shown to drive the loss of learning-induced striatal long-term potentiation (LTP) (Giordano et al., 2018). Therapeutic strategies focus on reducing α-syn-associated synaptic stress, preserving dopamine-dependent corticostriatal plasticity, and restoring neurotrophic support. However, direct causal evidence that complement-dependent synaptic pruning drives engram weakening in PD remains limited. Most studies report elevated complement transcripts or protein levels in the striatum, but functional interventions (e.g., blocking C1q or complement receptor 3) have not yet been tested for their effect on corticostriatal ensemble stability. Thus, current strategies are indirect disease-modifying approaches (e.g., AAV-mediated neurotrophic factor delivery to protect dopaminergic circuits) (Bäck et al., 2013). Their efficacy should be evaluated using engram-relevant outcomes such as restoration of learning-related LTP and task-linked ensemble coordination (Cheng et al., 2011; Yu et al., 2019; Ma et al., 2020).

#### 4.4.3 Huntington’s disease

ASO-mediated mHTT suppression reduces toxic mutant protein in all striatal and cortical cell types, rather than only striatal memory engram cells. Procedural memory recovery relies on widespread relief of cellular toxicity across neuronal and glial populations, a multifactorial dynamic outcome. In HD, upstream attenuation can directly target the causal driver mHTT. Intrathecal administration of the ASO tominersen (IONIS-HTTRx) in early-stage HD patients was well tolerated and produced dose-dependent reduction of mHTT in CSF, consistent with target engagement (Pal et al., 2006). Preclinical evidence further suggests stage dependence: in presymptomatic Q175 knock-in mice, ASO-mediated reduction of the mutant protein rescues activity-dependent BDNF secretion from the cortex to the striatum. However, this intervention is less effective once the animals exhibit symptoms (Park, 2018). These are indirect engram-relevant interventions because they lower cellular stress but do not directly prevent complement-mediated synaptic pruning (although complement proteins are upregulated around striatal synapses in HD models) (Wilton et al., 2023). Direct causal evidence that complement inhibition rescues ensemble stability in HD remains to be established.

Across various diseases, the examples align with three recurring priorities: reducing pathological load, preserving synaptic plasticity that supports engrams, and restoring circuit coordination. These priorities can be associated with circuit-level delivery and engram-based endpoints, as described below.

### 4.5 Translation: delivery and engram-based endpoints

All delivery systems and combinatorial therapeutic regimens described below achieve broad tissue coverage and affect diverse neural cell populations. No single intervention functions solely via modifying engram cells; all cognitive and pathological improvements originate from multifactorial interactions between general neural cell protection and engram ensemble stabilization.

Translational implementation necessitates three essential components: (i) targeted delivery to engram-enriched circuits, (ii) measurable engram-relevant readouts, and (iii) trial designs capable of detecting circuit-level changes earlier than broad cognitive scales.

Targeted delivery. Regionally targeted delivery is crucial for ensuring that therapies effectively reach memory-relevant circuits at adequate concentrations. For instance, focused ultrasound (FUS)-mediated region-targeted delivery of intravenous immunoglobulin (IVIg) to the hippocampus in AD mouse models has demonstrated improved blood-brain barrier (BBB) penetration and enhanced hippocampal exposure while preserving the effects related to Aβ plaques (Dubey et al., 2020). In PD models, AAV-mediated targeted delivery of neurotrophic factors has been shown to protect dopaminergic circuits (Bäck et al., 2013). In HD, both intrathecal and intranasal administration have facilitated the delivery of ASOs, resulting in measurable suppression of pathogenic products in both model and clinical contexts (Lin et al., 2021; Aly et al., 2023; Tabrizi et al., 2019). Overall, delivery strategies should be matched to circuits enriched for vulnerable engrams (hippocampal/entorhinal-hippocampal in AD; corticostriatal/striatal in PD; striatocortical in HD) rather than relying on systemic exposure alone.

Engram-based endpoints and trial readouts. Endpoints that directly reflect circuit integrity and memory-network performance enhance sensitivity for detecting disease-modifying effects; fMRI connectivity measures, spatial navigation tasks and circuit imaging jointly quantify global neural network integrity alongside engram-specific ensemble function, to disentangle contributions from pan-cellular neuroprotection versus selective engram rescue. Relevant fMRI metrics include altered hippocampal-PCC connectivity in AD, while virtual-reality spatial navigation tasks and focused ultrasound paradigms can evaluate hippocampal circuit integrity and memory connectivity changes in patients (Wu et al., 2011; Hohenfeld et al., 2018; Sánchez-Escudero et al., 2025; Jeong et al., 2025). These measures complement behavioral outcomes by providing objective assessments of circuit reinstatement capacity.

Human mechanistic decoding to inform precision combinations is essential. A critical need across diseases is the identification of druggable human signatures that indicate both compensation and vulnerability. Single-cell omics derived from post-mortem tissue or iPSC (Induced Pluripotent Stem Cell)-derived neurons can delineate disease-stage-specific molecular programs within vulnerable ensembles, thereby potentially guiding rational combination strategies and biomarker selection (Dai and Shen, 2022; Liu et al., 2024; Gutierrez Reyes et al., 2024; Rao-Ruiz et al., 2019). This aspect connects mechanistic engram biology to practical clinical readouts and target selection.

### 4.6 Summary: a multi-layer, molecule-to-circuit paradigm shift

Crucially, every therapeutic approach summarized below produces widespread regulatory effects on all central neural cell populations rather than acting selectively upon memory engram cells alone. The mitigation of neurodegenerative deficits is a multifactorial dynamic process integrating global neuroprotection and engram-specific functional rescue, with engram improvement representing only one contributing factor to overall clinical benefit. As outlined in Figure 3, current strategies converge on an integrated, multi-layer framework for engram-centered therapy that spans direct synaptic and circuit interventions, network-level coordination, and indirect disease modification.

At the most direct level, strengthening silent engram synapses—first demonstrated optogenetically in early AD models and now approximated non-invasively in humans via DTI-guided paired associative stimulation—offers a route to actively restore memory retrieval. Complement inhibition (e.g., C1q knockout in AD models) provides another direct strategy by selectively preventing the pruning of engram-relevant synapses, although equivalent causal evidence remains limited in PD and correlative in HD. Looking forward, epigenetic editing of engram cells may further stabilize memory persistence. At the network level, non-invasive approaches such as 40 Hz auditory gamma entrainment can restore default mode network connectivity, while targeting neocortical engrams could address remote memory deficits; these are indirect but clinically scalable means of facilitating ensemble reinstatement. Finally, disease-modifying strategies that reduce upstream pathology (Aβ, α-syn, mHTT) or provide neurotrophic support remain valuable but act indirectly by creating a permissive environment for engram function, with the important caveat that their effectiveness depends on early intervention, as highlighted by stage-dependent outcomes in HD models. Together, this molecule-to-circuit, multi-target paradigm marks a shift from symptomatic relief or broad neuroprotection toward the fundamental restoration of memory engram function, while acknowledging that the strength of evidence for direct complement-mediated pruning is strongest in AD, correlative in PD, and limited to early-stage models in HD. Notably, all proposed therapeutic interventions exert broad effects across diverse neural cell populations rather than exclusively targeting engram cells, and disease amelioration relies on multifactorial dynamic cellular crosstalk.

## 5 The rodent dependency problem, methodological heterogeneity and cross-species translational challenges in memory engram research

Although research on memory engrams has yielded groundbreaking insights into the neural basis of memory, its core conclusions are increasingly challenged by methodological heterogeneity and species-specific translational barriers. Critically, the vast majority of these findings are derived from rodent models, whose neural architecture, circuit organization, and behavioral repertoires differ substantially from those of primates and humans. This heavy reliance on a single taxonomic group raises fundamental questions about the generalizability of engram-based memory principles across species.

The limitations of the engram framework in rodent models can be summarized across three dimensions. First, causal validation relies heavily on invasive techniques such as optogenetics, which, despite their precision in targeting neuronal ensembles, face substantial barriers to direct translation to human studies (Pang et al., 2023; Jou et al., 2023; Josselyn and Tonegawa, 2020). Non-invasive repetitive transcranial magnetic stimulation (rTMS), while clinically promising, lacks the spatial resolution required to probe discrete engram cell function (Zoicas et al., 2024). Second, the mouse cortex exhibits a dense, “all-to-all” connectivity pattern, whereas primate and human cortices are organized into modular hierarchical architectures (Magrou et al., 2024; Roy et al., 2022; Takehara-Nishiuchi, 2021). Moreover, the extended maturation of the human prefrontal cortex and the intricate binding of what-where-when information far exceed the complexity observed in rodents, cautioning against direct extrapolation of rodent-based memory mechanisms to humans (Bevandiæ et al., 2024). Third, commonly used behavioral paradigms such as contextual fear conditioning are task-specific and engage neural circuits distinct from those underlying non-affective episodic-like memories, limiting their utility as sole proxies for general memory mechanisms (Hernández-Mercado and Zepeda, 2021). Collectively, these limitations underscore the inherent risks of cross-species extrapolation from the engram framework. Future research should prioritize the integration of cross-species comparative approaches and the development of non-invasive technologies to bridge the translational gap between micro-level mechanisms and macro-level cognition.

Mainstream engram studies typically rely on immediate early genes (IEGs), such as c-Fos, Arc, and Npas4, to label learning-activated neurons. Recent empirical evidence shows that these three IEGs exhibit markedly different co-expression patterns in neural circuits underlying aversive versus appetitive experiences, suggesting that a single IEG marker may be insufficient to fully capture the features of engram cells. Consequently, single-IEG labeling strategies may systematically omit or overrepresent specific functional subpopulations, biasing estimates of engram cell number and type (Arai et al., 2025).

This heterogeneity is further reflected in the differential responsiveness across neuronal subtypes. Distinct subtypes of inhibitory interneurons display highly heterogeneous IEG induction responses to stimuli; some subtypes (e.g., PV^+^) show low responses, whereas others (e.g., SOM^+^, VIP^+^) can exhibit strong IEG expression. Thus, in conventional IEG fluorescence-based experiments, the potential contribution of certain inhibitory interneuron subtypes (especially PV^+^ interneurons) to the engram is easily underestimated. Given the critical role of inhibitory circuits in regulating network dynamics, plasticity, and memory precision, this methodological blind spot directly leads to an incomplete understanding of the cellular composition of engrams across different brain regions (Giorgi and Marinelli, 2021).

The temporal dynamics and regional heterogeneity of IEG systems introduce additional methodological considerations. IEG transcription and translation typically occur within a narrow time window following a learning event, with protein expression peaking approximately 30 min after stimulation and declining over 120–240 min. However, different IEG promoters (e.g., c-Fos vs. Arc) exhibit significant differences in activation efficiency and recombination frequency across brain regions. For example, ArcTRAP mice show higher recombination efficiency than FosTRAP mice in most brain regions, whereas FosTRAP is more effective in the cerebellum and thalamus. This implies that engram identification based on a single IEG marker may be influenced by brain-region- and IEG-type-specific effects, leading to incomplete or biased depictions of engram cell populations (Pang et al., 2023).

These methodological differences fundamentally challenge the traditional view of engram cells as a homogeneous population. Therefore, future studies should employ single-cell-resolution transcriptomic analyses combined with multi-omics techniques (e.g., RNA-seq, ATAC-seq, Hi-C) to construct a more comprehensive and accurate molecular and cellular atlas of memory engrams (Yagishita and Sasaki, 2025).

Optogenetics is a core tool in engram research, but its clinical application faces multiple obstacles. A primary challenge is the dual requirement for gene therapy and implanted devices when applying optogenetics to the human brain. This technology necessitates both gene delivery (to express light-sensitive protein genes in specific neurons) and implantation of optical devices (to deliver light to targeted brain regions). Regarding gene therapy, the long-term safety of adeno-associated virus (AAV) vectors is a central concern; some studies have reported clonal expansion and vector integration in animal models of AAV therapy (Venditti, 2021). In addition, gene therapy faces clinical hurdles such as immunogenicity, limited targeting efficiency, and off-target distribution (Li et al., 2025). Concerning optical tools, the choice of light wavelength is critical because it directly affects tissue penetration. Effective light delivery to deep brain structures typically requires implantable optical fibers or endoscopes (Pang et al., 2023). Implanted fibers also carry risks of infection and inflammation. Biocompatibility of chronic implants is another major challenge, as devices must elicit low inflammatory responses (Lucchesi et al., 2025).

Despite these barriers to direct application, the primary contribution of optogenetics lies in its indirect translational pathway. A 2025 roadmap published in Nature Neuroscience clearly states that optogenetics will contribute to clinical practice not mainly through direct application in humans, but rather “by developing and advancing other therapeutic modalities using causal knowledge derived from optogenetic circuit neuroscience” (Lüscher et al., 2025). The roadmap further emphasizes that many translational paths do not depend on direct human application of optogenetics, but instead leverage this causal knowledge to advance other treatments. In other words, optogenetics is currently better suited as a basic discovery tool to unravel disease mechanisms rather than as a direct clinical therapeutic approach.

The complexity of neural circuits presents a further challenge for developing targeted therapies. When designing such therapies, one must consider the intricate organization of mammalian neural circuits and the extensive interconnectivity within the brain, because conventional assumptions about therapeutic targets may be biased by insufficient information (Lüscher et al., 2025). Researchers must also account for marked differences in brain volume and complexity between humans and other species (Lucchesi et al., 2025). These difficulties are compounded by ethical and safety concerns that are unavoidable in the clinical translation of optogenetics. Optogenetics involves writing, activating, erasing, or even manipulating memories, raising profound neuroethical issues in human applications—including threats to personal identity, autonomy, the long-term consequences of memory intervention, and potential alterations to an individual’s evaluative schemas and moral responses. Moreover, optogenetic techniques typically require delivery of exogenous genes into the brain, whose long-term expression and safety profiles remain incompletely understood. Manipulating memory traces or emotional states may disrupt the continuity and authenticity of personal identity, and may affect free will and moral responsibility (Adamczyk and Zawadzki, 2020). These combined challenges underscore that the successful translation of optogenetic and related circuit-based therapies will require not only neurobiological and technological advances, but also careful ethical scrutiny and regulatory frameworks.

## 6 Conclusion and future perspectives

This review connects engram biology to memory decline in AD, PD, and HD, systematically organizing evidence at the molecular, synaptic, and circuit levels. Crucially, all therapeutic strategies outlined herein do not exert exclusive effects on engram cells; improvements in neurodegenerative phenotypes stem from broad pan-cellular regulation and multifactorial dynamic interactions across neurons and glia. Acknowledging this multifactorial nature is essential, as it reframes engram-centered therapy not as selective ensemble manipulation, but as a strategy that restores the broader neural-glial ecosystem to enable engram function. Among these three diseases, AD offers the most complete experimental validation for our proposed engram-vulnerability framework, whereas mechanistic links in PD and HD remain largely inferential due to the scarcity of direct engram-targeted research. A prominent theme is the excitability-vulnerability tradeoff: engram ensembles necessitate high excitability, synaptic plasticity, and coordinated network activity to facilitate memory, yet these same requirements heighten sensitivity to synaptic stress, dysrhythmia, and inflammatory disturbances. Distinct pathological drivers, including Aβ/tau, α-syn associated with dopaminergic dysfunction, and mHTT, preferentially disrupt specific engram-enriched circuits, thereby elucidating the varying profiles of memory impairment observed across these disorders.

Immune-linked synaptic remodeling represents a common pathway from pathology to engram failure across various diseases. Chronic microglial activation and complement-mediated pruning can diminish engram-relevant connectivity and hinder cue-driven ensemble reinstatement. Furthermore, emerging evidence suggests a multicellular perspective in which astrocyte ensembles interact with neuronal engrams to influence recall. These convergent mechanisms indicate a practical translational opportunity: memory deficits may arise from compromised access and circuit coordination prior to significant neuronal loss. This situation allows for the possibility of monitoring and targeting synaptic integrity, plasticity capacity, and network coordination as immediate determinants of memory performance.

In conclusion, engram ensembles provide a coherent framework that links disease-specific pathology to circuit-level dysfunction and quantifiable memory outcomes. Future advancements will rely on the establishment of standardized engram-relevant endpoints, enhancements in non-invasive monitoring of memory-circuit function in humans, and the development of integrated neuronal-glial atlases that delineate molecular programs associated with reduced accessibility (“silent engrams”), synaptic destabilization, and impaired coordination. Critically, future trial designs should distinguish between global neuroprotective effects and engram-specific functional rescue, recognizing that combinatorial regimens—such as initiating pathology-lowering therapy followed by circuit-targeted neuromodulation—may be required to address the multifactorial nature of engram dysfunction. Collectively, these developments are expected to bolster mechanism-informed trial design and expedite the translation of engram-centered strategies aimed at preserving memory in neurodegenerative diseases.
